# Bisphenol A: Unveiling Its Role in Glioma Progression and Tumor Growth

**DOI:** 10.3390/ijms25052504

**Published:** 2024-02-21

**Authors:** Liang Niu, Juan Jia, Hu Yang, Shangyu Liu, Hongyu Wang, Yunji Yan, Qiao Li, Qiang Dong, He Zhang, Guoming Zhao, Junqiang Dai, Guoqiang Yuan, Yawen Pan

**Affiliations:** 1 Lanzhou University Second Hospital, The Second Medical College of Lanzhou University, Cuiyingmen No. 82, Chengguan District, Lanzhou 730030, China; niul20@lzu.edu.cn (L.N.); jiaj20@lzu.edu.cn (J.J.); liushy93@126.com (S.L.); wanghy20@lzu.edu.cn (H.W.); yanyj20@lzu.edu.cn (Y.Y.); starks3@163.com (Q.L.); zhangh20@lzu.edu.cn (H.Z.); zhaogm20@lzu.edu.cn (G.Z.); daijunqiang91@sina.com (J.D.); 2 Department of Neurosurgery, Second Hospital of Lanzhou University, Lanzhou 730030, China; yanghu8163@163.com (H.Y.); dongq19@lzu.edu.cn (Q.D.); 3 Department of Anesthesiology, Second Hospital of Lanzhou University, Lanzhou 730030, China; 4 Key Laboratory of Neurology of Gansu Province, Lanzhou University, Lanzhou 730030, China

**Keywords:** bisphenol A (BPA), glioma, cell proliferation, melatonin

## Abstract

Gliomas represent the most common and lethal category of primary brain tumors. Bisphenol A (BPA), a widely recognized endocrine disruptor, has been implicated in the progression of cancer. Despite its established links to various cancers, the association between BPA and glioma progression remains to be clearly defined. This study aimed to shed light on the impact of BPA on glioma cell proliferation and overall tumor progression. Our results demonstrate that BPA significantly accelerates glioma cell proliferation in a time- and dose-dependent manner. Furthermore, BPA has been found to enhance the invasive and migratory capabilities of glioma cells, potentially promoting epithelial–mesenchymal transition (EMT) characteristics within these tumors. Employing bioinformatics approaches, we devised a risk assessment model to gauge the potential glioma hazards associated with BPA exposure. Our comprehensive analysis revealed that BPA not only facilitates glioma invasion and migration but also inhibits apoptotic processes. In summary, our study offers valuable insights into the mechanisms by which BPA may promote tumorigenesis in gliomas, contributing to the understanding of its broader implications in oncology.

## 1. Introduction

Gliomas constitute approximately 80% of primary malignant brain tumors, embodying a complex and intricate biology. Despite extensive research efforts over the years, many facets of glioma biology continue to be shrouded in uncertainty [1]. Glioblastoma, the most common and aggressive variant of glioma, is distinguished by its characteristic central necrosis and pronounced microvascular proliferation leading to rapid, highly invasive growth and significantly low survival rates [2]. In this context, the field of glioma epidemiology is vigorously pursuing the identification of modifiable risk factors to aid in the prevention and management of this formidable disease. To date, the only confirmed risk factors for glioma development are high-dose ionizing radiation exposure and specific genetic mutations linked to rare hereditary syndromes [3]. Recent scientific endeavors have pivoted towards the identification of genetic variants associated with increased glioma susceptibility, as well as the utilization of molecular markers to categorize glial tumors into more homogenous and clinically relevant subsets. Given the intricate interplay between gene products, environmental exposures, and developmental signals in the pathogenesis of gliomas, it is imperative that further comprehensive studies are undertaken to elucidate the relationships between polymorphisms and glioma incidence. A deeper understanding of glioma cell motility and its underlying biology is paramount in the pursuit of innovative therapeutic strategies to effectively combat this disease.

Bisphenol A (BPA) is a widely used chemical in the manufacturing of various plastic products, including water bottles and food containers, as well as in the linings of canned foods [4]. Recognized as an endocrine disruptor, BPA can interfere with the body’s hormonal systems. Alarmingly, the prevalence of various cancers, such as those of the breast, ovary, uterus, prostate, and testes, has been surging, with some attributions given to BPA exposure [5,6,7,8,9]. Beyond BPA, pesticides have also been implicated in the development of gliomas [10]. Observational studies suggest a heightened risk of gliomas among individuals engaged in agriculture or residing in areas with substantial pesticide utilization [11]. Further inquiries into glioma etiology have investigated the roles of solvents, heavy metals, and electromagnetic fields [12]. Additionally, animal model studies indicate that BPA exposure can induce significant alterations in the brain and nervous system, encompassing changes in brain structure and function [13,14].

It is critical to acknowledge that the evidence linking chemical exposures, including BPA, to glioma development remains inconclusive. While some studies suggest a potential association between BPA exposure and brain tumor formation, including gliomas, conclusive evidence establishing a direct relationship is still lacking. This uncertainty underscores the necessity for further research to elucidate the possible implications of BPA for glioma development comprehensively.

In our study, we explored the potential promotional effects of BPA on glioma proliferation. Utilizing bioinformatics approaches, we evaluated the associated risks of BPA in a glioma cohort and developed a risk assessment model accordingly. Our findings suggest that BPA contributes to the enhancement of glioma invasion and migration and appears to suppress apoptosis. Additionally, our results imply that BPA might influence the epithelial–mesenchymal transition (EMT) properties of glioma cells, warranting further investigation into its role in tumor progression and malignancy.

## 2. Results

### 2.1. Examining the Interplay between Bisphenol-Associated Gene Pathways

**Figure 1:**
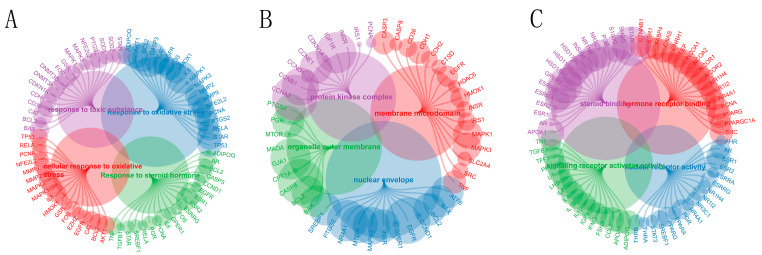
Gene ontology enrichment analysis of BPA-related genes: (**A**) This panel illustrates the significant enrichment of genes involved in the response to steroid hormones and oxidative stress. Colored connections represent the interaction between genes and their associated biological processes, highlighting the coordinated regulation of cellular responses to environmental stimuli. (**B**) Shown here are the cellular components enriched in genes that include nuclear receptor activity, steroid binding, and hormone receptor binding. The distribution of genes across these components suggests their collective role in signaling pathways and receptor-mediated mechanisms. (**C**) This section depicts the molecular functions and cellular components associated with membrane microdomains, protein kinase complexes, and the structures of organelles. Each arc illustrates the specific contribution of genes to the functional complexity of the cell’s architecture and signaling systems.

To elucidate the complex interactions between bisphenol A (BPA) and gene pathways, we initiated our study with an in-depth examination of the Comparative Toxicogenomics Database (CTD). We employed the ClusterProfiler package in R (version 4.0.3) to conduct a gene ontology (GO) enrichment analysis, aiming to unveil the intricate biological processes, molecular functions, and cellular components associated with genes differentially expressed in response to BPA. Our analysis revealed a notable enrichment in key biological processes, particularly those related to steroid hormone response, oxidative stress response, and cellular oxidative stress response (Figure 1A). Additionally, the implicated genes were significantly involved in molecular functions pertaining to membrane microdomains, protein kinase complexes, organelle outer membranes, and nuclear envelopes (Figure 1C), as well as in cellular components including nuclear receptor activity, steroid binding, hormone receptor binding, and signaling receptor activator activity (Figure 1B). Overall, the GO enrichment analysis provided a comprehensive understanding of the roles these differentially expressed genes play in various cellular contexts and their potential linkage to BPA exposure. 

### 2.2. Investigating the Role of Bisphenol-Related Compounds in a Glioma Cohort Using the ssGSEA Algorithm

**Figure 2:**
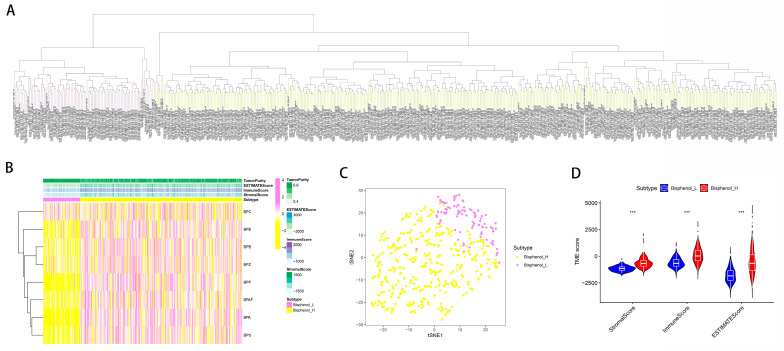
Bisphenol score analysis and correlation with tumor microenvironment in glioma. (**A**) This dendrogram illustrates the distribution of bisphenol-related scores across the glioma cohort. Scores were derived from ssGSEA analysis, showing the hierarchical clustering of patients based on their score magnitude. (**B**) The heatmap displays bisphenol-related scores across individual glioma patients. Each column represents a patient, with color intensity corresponding to the score magnitude, revealing the diversity of bisphenol’s influence within the cohort. (**C**) This bar chart divides the cohort into high and low bisphenol score groups. The bars represent the percentage of patients in each group, demonstrating the binary classification based on the ssGSEA-derived scores. (**D**) Violin plots illustrate the positive correlation between high bisphenol scores and immune, stromal, and ESTIMATE scores in glioma patients. The width of each plot indicates the density of data points at different score levels. *** = *p* ≤ 0.001.

**Figure 3:**
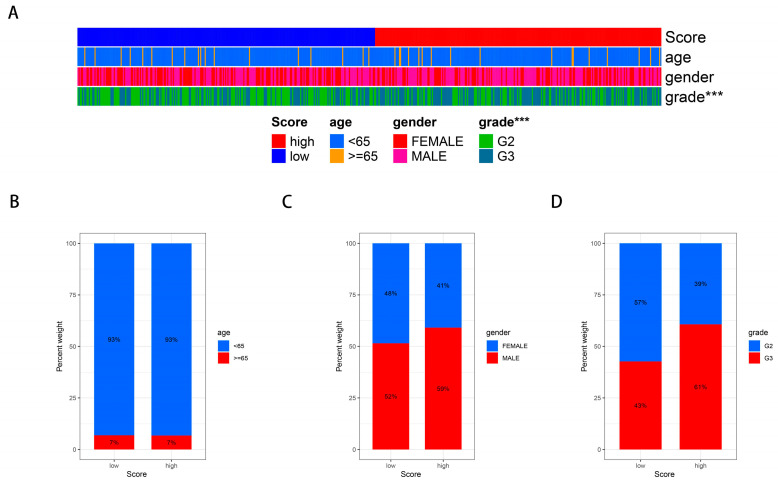
Association of bisphenol-related scores with glioma grade. (**A**) The bar plots represent the proportions of glioma patients with high and low bisphenol-related scores according to age, gender, and tumor grade. The colors denote different demographic and clinical characteristics, providing a clear visualization of their distribution across the scores. (**B**–**D**) These panels break down the proportions of patients with high and low bisphenol-related scores by age (**B**), gender (**C**), and tumor grade (**D**). The stacked bars show the percentages within each score category, revealing patterns of association with these key patient demographics and tumor characteristics. *** = *p* ≤ 0.001.

To evaluate the influence of bisphenol-related compounds within a glioma cohort, we employed the ssGSEA analysis to derive bisphenol-related scores (Figure 2A). The cohort was then divided into two groups characterized by high and low bisphenol scores (Figure 2C). A heatmap effectively illustrated the distribution of bisphenol-related scores among glioma patients, providing a visual representation of variance across the cohort (Figure 2B). Furthermore, we explored the correlation between bisphenol-related scores and the tumor microenvironment (TME). Intriguingly, a positive correlation emerged, linking higher bisphenol scores with increased immune, stromal, and ESTIMATE scores in glioma patients, suggesting an interaction between bisphenol exposure and tumor microenvironment dynamics (Figure 2D). Additionally, a relationship was observed between elevated bisphenol-related scores and more advanced tumor grades (Figure 3), indicating potential implications regarding tumor aggressiveness and prognosis.

### 2.3. Developing a BPA-Related Risk Model in a Glioma Cohort

**Figure 4:**
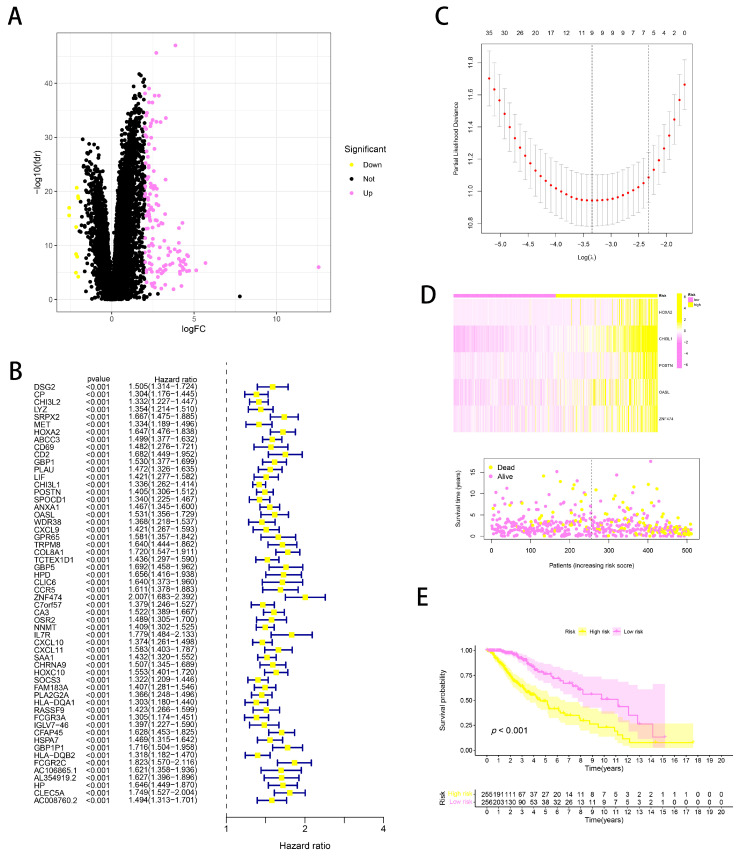
Prognostic gene identification and risk stratification in glioma based on BPA association. (**A**) Volcano plot displaying the differentially expressed genes between BPA-low and BPA-high groups in the glioma cohort. Genes are color-coded based on significance and direction of expression change, with key genes highlighted. (**B**) Forest plot illustrating hazard ratios for individual genes identified in the univariate COX regression analysis. Bars represent the 95% confidence intervals, indicating the prognostic impact of each gene on survival. (**C**) Profile plot showing the selection of optimal prognostic genes using LASSO regression. The plot traces the changes in the LASSO coefficients as the penalty parameter lambda is varied. (**D**) The top panel is a heatmap of the expression levels of key prognostic genes across the glioma cohort, with patients ordered by increasing risk score. The bottom panel is a scatter plot showing the distribution of survival times for patients, color-coded by risk category. (**E**) Kaplan–Meier curves comparing overall survival between low- and high-risk patient groups, with risk determined by the prognostic model. The shaded area represents the 95% confidence interval, and the number of patients at risk over time is listed below.

To discern BPA-related genes within the glioma cohort, we conducted a differential expression analysis contrasting the BPA-low and BPA-high groups. This revealed numerous genes with varied expression levels among the cohort (Figure 4A). Subsequent analyses included a univariate COX regression followed by a LASSO regression, which helped to identify genes related to prognosis (Figure 4B,C). We then implemented a multivariate COX regression analysis to create a prognostic prediction model that incorporated BPA features into the glioma context. Risk scores were assigned to each glioma patient based on a specific formula: risk score = HOXA20.158262352253815 + CHI3L10.163539741852195 + POSTN0.122461088150864 + OASL0.160406690819272 + ZNF474 × 0.333919193479214. These five genes emerged as significant contributors within the glioma cohort. Patients were then stratified into low- and high-risk categories based on median risk scores (Figure 4D), with higher scores correlating with diminished overall survival (OS) rates, as illustrated in Figure 4E. This comprehensive approach highlights the potential of BPA-related genetic markers in predicting glioma prognosis.

### 2.4. Correlating a BPA-Related Risk Model with Clinical Features and Constructing a Predictive Nomogram in a Glioma Cohort

**Figure 5:**
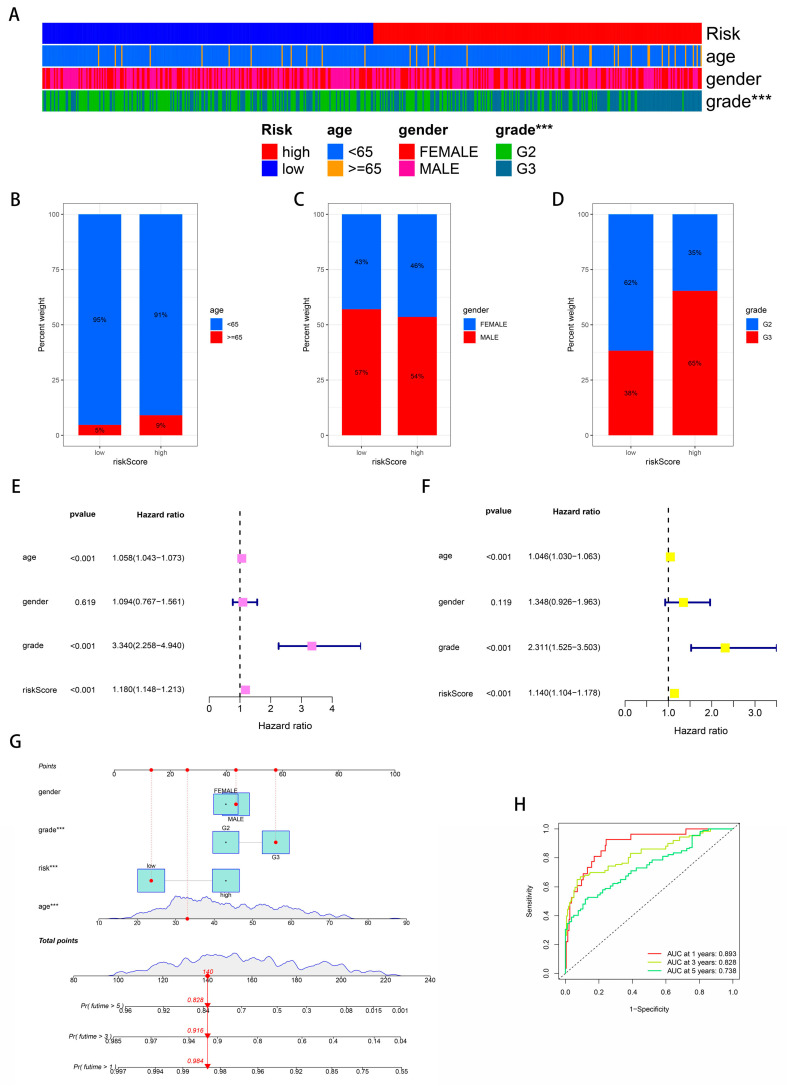
Clinical correlation and predictive performance of BPA-related risk model for glioma. (**A**) Bar chart summarizing the distribution of clinical features such as age, gender, and tumor grade across high and low BPA-related risk scores in the glioma cohort. (**B**–**D**) These bar charts compare the percentage of glioma patients with low and high BPA-related risk scores across different age groups (**B**), genders (**C**), and tumor grades (**D**), illustrating the correlation between risk scores and clinical features. (**E**) Forest plot displaying the hazard ratios for age, gender, tumor grade, and risk score derived from univariate analysis. Bars represent confidence intervals, indicating the strength of each factor’s association with patient prognosis. (**F**) Forest plot showing hazard ratios for the same clinical features from multivariate analyses, confirming their status as independent risk factors. (**G**) The nomogram integrates clinical features with the BPA-related risk scores to predict individual patient survival. Points are assigned for each variable, which correspond to a predicted survival probability at specific time points. (**H**) The receiver operating characteristic (ROC) curve evaluates the predictive accuracy of the BPA-based risk model over different time points. The area under the curve (AUC) values provide a measure of the model’s performance. *** = *p* ≤ 0.001.

Correlation analyses between BPA-related risk models and clinical characteristics were conducted, revealing that patients with higher risk scores often presented with more advanced diseases (Figure 5A–D). Age, tumor grade, and risk score emerged as independent prognostic factors within the glioma cohort, as demonstrated by both univariate and multivariate analyses (Figure 5E,F). To enhance their prognostic utility for glioma, we developed a nomogram incorporating clinical features such as gender, tumor grade, and age, aiming for superior predictive accuracy (Figure 5G). The performance of this BPA-based risk model was further affirmed by ROC curve analysis, showcasing its excellent predictive capacity in determining glioma outcomes (Figure 5H). These findings underscore the potential of integrating BPA-related risk scores with clinical parameters to refine prognosis and management strategies for glioma patients.

### 2.5. BPA Affects the Cell Viability of Glioma Cells in a Manner That Is Dependent on Its Concentration

**Figure 6:**
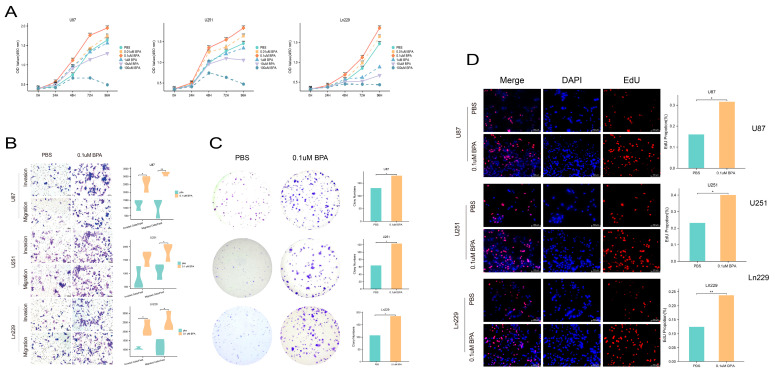
CCK8, Transwell, colony formation, and EdU experiments conducted on U87, U251, and Ln229 cell lines revealed that a concentration of 0.1 µM BPA stimulates the proliferation, migration, and invasion of glioma cells. (**A**) The CCK8 assay yielded findings that indicate BPA’s capacity to stimulate the proliferation of glioma cells, although it is noteworthy that a concentration of 100 µM exhibited a decelerating effect on growth. (**B**) The presence of 0.1 µM BPA enhances the invasion and migration of glioma cells. The (**C**) colony formation and (**D**) EdU assays both showed that 0.1 µM BPA significantly augmented the glioma cells’ aptitude for clone formation and proliferation. ns = *p* > 0.05, * = *p* ≤ 0.05, ** = *p* ≤ 0.01, *** = *p* ≤ 0.001.

To delineate the effects of bisphenol A (BPA) on malignant glioma cells, we exposed the U87, U251, and Ln229 cell lines to a range of BPA concentrations over a period of 0 to 96 h. A BPA concentration gradient from 0.01 µM to 100 µM was established to gauge its impact on cell viability. Using CCK8 assays, all three glioma cell lines showed similar responses to BPA exposure. Notably, cells treated with 0.01 µM, 0.1 µM, 1 µM, and 10 µM concentrations of BPA displayed increased viability compared to the PBS control group, with 0.1 µM showing the most pronounced effect on cell proliferation. Conversely, the 100 µM concentration demonstrated a deleterious effect, significantly inhibiting cell viability across the entire 0 to 96 h duration (Figure 6A). Consequently, we selected 0.1 µM BPA for subsequent experiments, omitting the 100 µM concentration due to its cytotoxicity and low relevance to typical environmental exposures. This approach allowed us to understand the nuanced effects of BPA on glioma cell dynamics and identify optimal conditions for further studies.

### 2.6. BPA Increased the Proliferation of Glioma Cells

The application of 0.1 µM BPA to the glioma cell lines U87, U251, and Ln229 resulted in a significant increase in proliferation, as evidenced by the marked rise in EdU-positive cells (Figure 6D). This observation suggests a notable enhancement of cell proliferation that is attributable to BPA exposure. Further exploration of BPA’s long-term effects was conducted through colony formation assays. Remarkably, after a 10-day incubation period with 0.1 μM BPA, all three glioma cell lines demonstrated substantial increases in colony formation, confirming the proliferation-promoting effects of BPA (Figure 6C). To monitor the proliferation dynamics of living cells continuously and comprehensively, high-content dynamic phase contrast (DPC) imaging was utilized, capturing sequential images of cell proliferation. The results unequivocally showed a significant and consistent increase in glioma cell proliferation upon BPA treatment, without any signs of cell shrinkage or death (Figure 6B). These collective findings robustly demonstrate that BPA, at a concentration of 0.1 μM, markedly promotes glioma cell proliferation, indicating its role as a substantial factor in tumor cell growth dynamics.

### 2.7. Bisphenol A Enhances the Migration and Invasion of Glioma Cells

**Figure 7:**
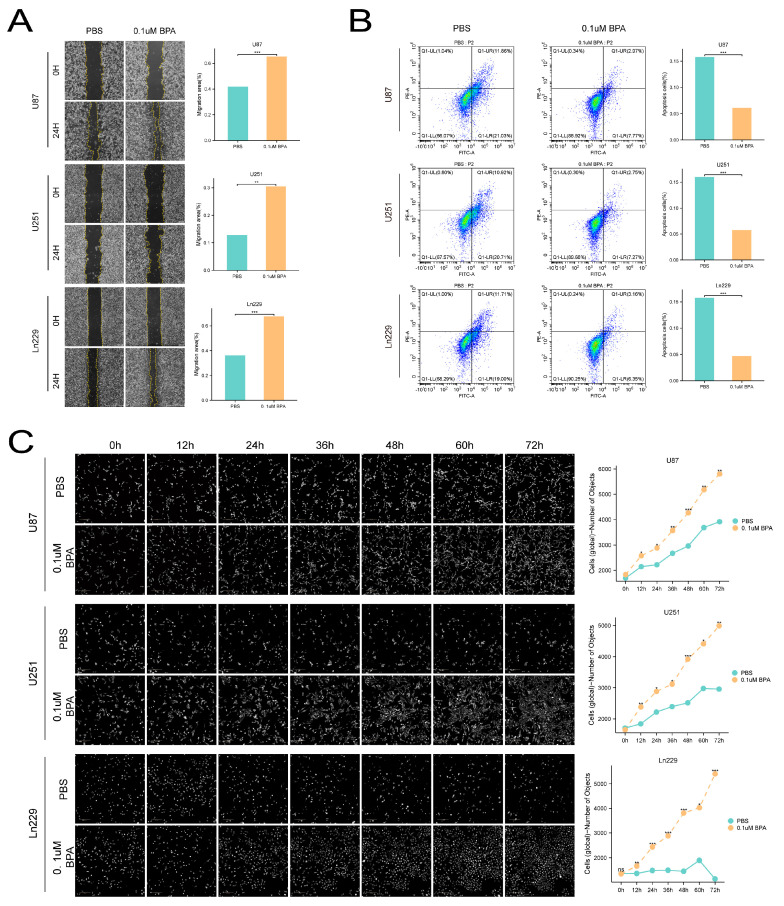
(**A**) The wound healing assay confirmed that a concentration of 0.1 µM BPA enhanced the migration of U87, U251, and Ln229 cells. (**B**) BPA also inhibited apoptosis in glioma cells compared to the control group that did not receive BPA treatment. (**C**) The proportion of early and late apoptosis was significantly decreased. High-content DPC analysis showed that BPA-treated glioma cells exhibited accelerated growth without any observable signs of apoptosis, such as nuclear fragmentation or cell death, throughout the growth process. ns = *p* > 0.05, * = *p* ≤ 0.05, ** = *p* ≤ 0.01, *** = *p* ≤ 0.001.

Following our comprehensive analysis of BPA’s effects on glioma cell viability in vitro, we extended our research to examine its impact on the metastatic behaviors of these cells. Using wound healing assays, we observed that a concentration of 0.1 µM BPA significantly stimulated the migration of U87, U251, and Ln229 cell lines (Figure 7A). Similarly, Matrigel invasion assays provided further evidence of BPA’s influence, revealing enhanced invasive capabilities in Ln229 cells alongside U87 and U251 cells (Figure 7B). These findings collectively indicate that both the invasion and migration of glioma cells were markedly increased following a 24 h treatment period with 0.1 µM BPA, underscoring its potential role in promoting metastatic characteristics in glioma cells.

### 2.8. BPA Inhibits the Apoptosis of Glioma Cells

To examine the potential connection between BPA-induced proliferation and apoptosis in glioma cells, we treated the U87, U251, and Ln229 cell lines with 0.1 µM BPA over a period of 48 h. Apoptosis levels were assessed using FITC Annexin V/PI staining. Our analysis revealed a significant decrease in both the early and late stages of apoptosis among the cells treated with BPA compared to the untreated control group. This reduction was particularly pronounced in the late apoptotic cells (Figure 7B), suggesting that BPA may contribute to glioma cell survival by mitigating apoptotic processes. These observations highlight the potential of BPA to influence the balance between cell proliferation and death, further implicating its role in the progression of glioma.

### 2.9. Melatonin Mitigates Bisphenol A-Induced Proliferation and Metastasis in Glioma Cell Lines

**Figure 8:**
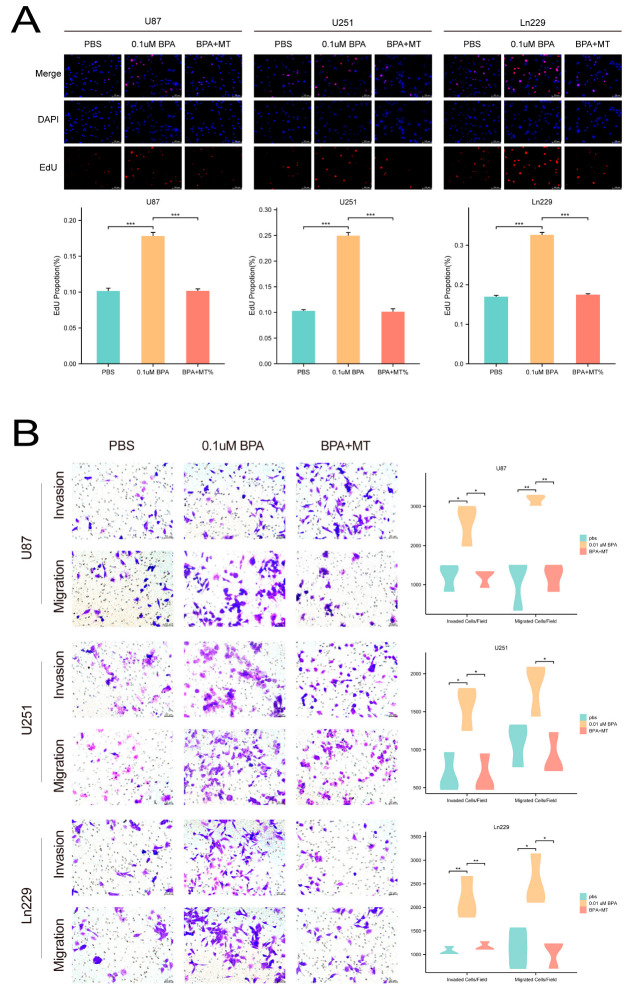
(**A**,**B**) Melatonin (MT) mitigated the effects of BPA. Co-treatment with melatonin notably decreased the proliferation rate of glioma cells and restricted their invasion and migration capabilities. * = *p* ≤ 0.05, ** = *p* ≤ 0.01, *** = *p* ≤ 0.001.

Based on the pathway analysis, we discovered significant enrichment in the oxidative stress pathway among glioma cells exposed to bisphenol A (BPA). To further explore the role of oxidative stress in BPA-induced glioma progression and potentially counteract its effects, we conducted rescue experiments using melatonin, a well-known oxidative stress inhibitor (Figure 8). In exploring the oncogenic effects of bisphenol A (BPA) on glioma cells, our investigation revealed that treatment with 0.1 µM BPA significantly promoted cell proliferation and metastatic behaviors in the U87, U251, and Ln229 cell lines, as evidenced by increased EdU incorporation and enhanced capabilities in invasion and migration assays. Notably, the addition of melatonin (MT), a known oxidative stress inhibitor, attenuated these BPA-induced effects. Co-treatment with melatonin resulted in a marked reduction in proliferation rates and restricted the invasive and migratory capacities of the glioma cells (Figure 8A,B). These results suggest that, while BPA exacerbates the aggressive traits of glioma cells, melatonin offers a potential counteractive approach, shedding light on its therapeutic promise in glioma management.

## 3. Discussion

Gliomas are a complex and often aggressive category of brain tumors arising from glial cells, which include astrocytes, oligodendrocytes, and ependymal cells. They are classified based on cell type, grade, and location within the brain [15]. Low-grade gliomas typically grow more slowly and have better prognoses, while high-grade gliomas, such as glioblastoma multiforme (GBM), are fast-growing and often lethal [16]. The symptoms of gliomas are diverse and depend largely on the tumor’s location and size, with common signs including headaches, seizures, and cognitive or neurological deficits. Treatment modalities vary from surgical resection to radiation therapy and chemotherapy, often combined in a multi-modal approach tailored to the individual patient’s needs and tumor characteristics [17]. Despite advances in neuroimaging, surgery, and adjuvant therapies, gliomas, particularly high-grade variants, continue to present significant treatment challenges due to their invasive nature and the brain’s sensitive and complex architecture [18]. The health impacts of BPA exposure have been the subject of extensive research, with ongoing debates about its potential adverse effects on humans, particularly at minimal exposure levels. Recent scientific literature has investigated the association between BPA exposure and cancer induction in humans and laboratory animals. Studies have demonstrated that even small quantities of BPA can disrupt cellular pathways and initiate molecular mechanisms related to cell proliferation and chemotherapy resistance [19]. The biological activity of BPA is reportedly higher in humans than in mice at similar exposure levels [20]. BPA has been detected in various bodily fluids and tissues, including saliva, serum, urine, amniotic fluid, follicular fluid, placental tissue, and breast milk [21]. Its accumulation in adipose tissue and persistent activity due to its short half-life lead to increased BPA levels in the bloodstream over time [22]. Additionally, BPA exposure has been associated with enhanced N-ras expression in K. marmortatus, suggesting increased vulnerability to environmental carcinogenesis [23].

Research has established a clear link between BPA exposure and an elevated incidence of certain cancers. Specifically, studies have shown that individuals with higher concentrations of BPA in their urine have a significantly increased risk of developing meningiomas, with a 1.4 to 1.6 times higher likelihood compared to those with lower BPA levels [24]. Our findings contribute to the growing body of evidence suggesting a correlation between BPA exposure and brain tumor development, underscoring the need for continued investigation into the potential health implications of BPA.

Considering the potential mechanisms involved, we selected three glioma cell lines for in vitro studies. In the CCK8 assays, glioma cell viability and proliferation exhibited significant enhancement in response to various BPA concentrations (0.01 µM, 0.1 µM, 1 µM, and 10 µM) in a dose- and time-dependent manner. Notably, a 100 µM concentration of BPA displayed inhibitory effects. These observations align with prior studies indicating the cytotoxic effects of high concentrations of BPA on various cell types, including neural progenitor, cervical, and colon cancer cells [25,26,27]. Echoing the findings of Wang et al. [28] and Shi et al. [29], our study similarly identified an inverted U-shaped dose–response curve, observing inhibitory effects with 100 µM BPA. While exposure to such high concentrations is unlikely in environmental settings, the most pronounced effects in our study occurred with 0.1 µM BPA. Consequently, this concentration was chosen for further investigation to progressively elucidate its effects.

Acknowledging BPA’s pervasive presence and its time-dependent effect on cellular proliferation, we extended the exposure duration in our study to 96 h, surpassing the typical 48 to 72 h timeframe utilized in previous research. Tumor progression encompasses several critical processes, including proliferation, migration, invasion, and cell death, with migration and invasion being particularly crucial in tumor metastasis. The epithelial-to-mesenchymal transition (EMT) is vital for metastatic spread, characterized by reduced cellular adhesion and increased mesenchymal traits, facilitating cancer cell dissemination [30]. Our EdU proliferation assays confirm that BPA significantly promotes cancer cell proliferation. BPA has been shown to regulate gene expression related to cell migration, thereby enhancing EMT. It specifically increases the expression of proteins such as the waviness protein, cathepsin D, and matrix metalloproteinase-2 (MMP-2), while decreasing E-cadherin expression [31]. MMPs, crucial for extracellular matrix degradation and the modification of its structure, are instrumental in cancer cell invasion. Our wound healing assays corroborate that BPA increases cell migration, providing insight into its potential role in glioma progression. The Transwell assay further supported the notion that BPA may augment glioma cell invasion and migration, indicative of its influence on EMT characteristics. These findings collectively underscore the potential role of BPA in promoting the invasive and metastatic properties of glioma cells.

Apoptosis and cell cycle arrest are pivotal mechanisms in inhibiting cell proliferation. In our study, we aimed to identify markers of apoptosis in glioma cells following BPA exposure. Utilizing flow cytometry, we found that BPA notably impedes the onset of apoptosis in glioma cells, particularly in the advanced stages. Such alterations typically involve the induction of inflammatory responses, which subsequently attract immune cells and lead to the production of pro-carcinogenic cytokines. These cytokines then activate signaling pathways that enhance cell proliferation and suppress apoptosis [32,33,34]. Further, we investigated BPA’s impact on glioma cell morphology using high-content screening dynamic phase contrast (DPC). The results indicated a marked increase in the growth of BPA-treated glioma cells, with no visible signs of apoptosis such as pronounced nuclear condensation or fragmentation throughout the observation period.

To deepen our understanding of BPA’s potential mechanisms in glioma, this study conducted a comprehensive analysis of the risks associated with bisphenol A in a glioma cohort. Our primary aim was to pinpoint genes that were uniquely responsive to bisphenol exposure within this group. Through rigorous differential expression analysis between bisphenol-low and bisphenol-high groups, we observed significant variations in gene expression across the cohort. We identified five critical genes (HOXA2, CHI3L1, POSTN, OASL, and ZNF474) with substantial roles in the glioma context. These genes facilitated the classification of patients into low- and high-risk categories based on their gene expression profiles, thereby offering a nuanced approach to patient stratification. This study lays substantial groundwork for future research aimed at uncovering the intricate links between BPA exposure and specific gene expressions in glioma.

In our findings, the observed protective role of higher concentrations of bisphenol A against glioma migration presents an intriguing aspect, notably due to the limited direct explanations available in the current literature. We hypothesize that this phenomenon could be attributed to a non-linear pharmacological response, where elevated concentrations of bisphenol A initiate alternate cellular mechanisms, potentially involving cellular toxicity or adaptive stress responses that mitigate migration. Additionally, it is plausible that, at higher concentrations, bisphenol A interacts with cellular components or pathways not primarily associated with its known effects, leading to altered glioma cell migration behaviors. These interpretations are preliminary and highlight the complexity of bisphenol A’s biological effects, underlining the necessity for further research. Consequently, mechanistic studies and detailed explorations of molecular pathways are imperative in order to elucidate the underlying mechanisms. We recognize the need for additional research and will seek to explore these avenues in future studies, thereby contributing to a more comprehensive understanding of bisphenol A’s role in glioma migration.

Our study has laid a foundational understanding of the potential relationship between BPA exposure and glioma development, highlighting the complex interplay between environmental factors and cancer biology. However, several avenues remain unexplored and warrant further investigation. Firstly, longitudinal studies are necessary to elucidate the long-term effects of BPA exposure on glioma progression and patient prognosis. Such studies could provide more definitive evidence regarding the causality and magnitude of BPA’s impact on glioma development. Secondly, exploring the genetic and epigenetic modifications induced by BPA exposure in glioma cells could reveal the molecular mechanisms underlying BPA’s effects. This includes detailed analyses of DNA methylation patterns, histone modifications, and non-coding RNA expression profiles in response to BPA. Thirdly, considering the variability in individual susceptibility to BPA, research into genetic polymorphisms that may influence BPA metabolism and action could offer insights into personalized risk assessments and interventions. Finally, alternative bisphenols and their derivatives, which are increasingly used as BPA substitutes, also merit investigation to assess their safety and potential carcinogenic effects in the context of glioma and other cancers.

While our study provides significant insights, it is not without limitations. The in vitro nature of our experiments, although informative, may not fully replicate the complex in vivo environment where multiple factors interact simultaneously. Therefore, the extrapolation of our findings to clinical scenarios should be approached with caution. Additionally, the concentrations of BPA used in our experiments, particularly the higher doses, may not accurately reflect typical human exposure levels, potentially limiting the relevance of our findings to environmental exposure scenarios. Our study also did not account for the potential synergistic effects of BPA with other environmental contaminants, which could collectively influence glioma development and progression. Another limitation is the reliance on a limited number of glioma cell lines, which may not capture the full genetic and phenotypic diversity of gliomas in patients. This could affect the generalizability of our findings across different glioma subtypes and grades.

## 4. Materials and Methods

### 4.1. Acquisition of the Dataset

To investigate the genes interacting with bisphenol A (BPA), we utilized data from the Comparative Toxicogenomics Database (CTD), available at http://ctdbase.org/ (accessed on 15 May 2023). This database provided us with a comprehensive list of genes known to interact with BPA, forming the basis of our investigation. Additionally, we acquired mRNA expression data of glioma patients from The Cancer Genome Atlas (TCGA) database, accessible at https://portal.gdc.cancer.gov/ (accessed on 15 May 2023). Specifically, we focused on the Glioblastoma Multiforme (GBM) cohort within TCGA, which comprises extensive genomic data pertinent to our study on gliomas. The integration of these databases allowed us to correlate the BPA-interacting genes with the expression profiles observed in glioma patients, facilitating a multifaceted analysis of BPA’s potential impact on glioma pathogenesis.

### 4.2. Differential Expression Analysis

To explore the impact of BPA on glioma, we extracted RNA sequencing data along with pertinent clinical information from the Glioblastoma Multiforme (GBM) cohort of The Cancer Genome Atlas (TCGA) dataset. Our focus was specifically on analyzing the differential expression of mRNA in glioma patients in relation to their BPA exposure profiles. For the processing and analysis of these data, we employed the Limma package in R (version 4.0.3), a robust tool for the analysis of gene expression data arising from microarray or RNA-seq technologies. Differential expression analysis was conducted to identify significant changes in mRNA levels between BPA-exposed and unexposed groups in the glioma cohort. We set stringent criteria for identifying significantly differentially expressed genes, requiring a *p*-value of less than 0.05 combined with a log2 fold change greater than 0.585 for upregulated genes or less than −0.585 for downregulated genes.

### 4.3. Pathway Enrichment Analysis

To elucidate the potential functions and biological pathways associated with the target genes implicated by bisphenol A (BPA) exposure in gliomas, we conducted functional enrichment analysis. Gene ontology (GO) annotations were utilized as a foundational method to categorize and denote the molecular functions (MF), biological processes (BP), and cellular components (CC) associated with the differentially expressed genes. Specifically, we employed the ClusterProfiler package in R (version 4.0.3), a powerful tool for comparing and visualizing functional profiles among gene clusters. This allowed us to perform both GO enrichment and Kyoto Encyclopedia of Genes and Genomes (KEGG) pathway analyses. KEGG analysis provided a comprehensive understanding of the high-level functions and utilities of the biological system, such as the cell, organism, and ecosystem, based on genomic information. Through the integration of GO annotations and KEGG pathway analysis, we systematically analyzed the functions and interactions of potential mRNA targets. This approach enabled us to not only decipher the molecular and cellular context in which these genes operate but also identify specific genes with oncogenic potential or involvement in cancer-related pathways. The identification and understanding of these enriched pathways and target genes provide valuable insights into the molecular mechanisms of glioma progression and the potential impact of BPA on these processes.

### 4.4. Protein–Protein Interaction (PPI) Network Construction

To investigate the potential internal correlations and interactive networks among proteins encoded by genes implicated in BPA-related glioma progression, we utilized the STRING database (https://cn.string-db.org/ (accessed on 15 May 2023)). STRING is a comprehensive resource that compiles protein–protein interaction data from a variety of sources, including high-throughput lab experiments, computational predictions, consolidated genomic context predictions, co-expression data, and findings from relevant scientific literature through automated text mining. We extracted detailed information regarding the proteins and their known or predicted interactions relevant to our study’s gene set. The interactions encompassed both physical and functional associations, providing a broad understanding of the connectivity and potential coordination among the proteins of interest. For the visualization and further analysis of these protein–protein interaction networks, we employed Cytoscape, an open-source bioinformatics software platform R (version 4.0.3) designed for the visual integration of network data with high-dimensional expression data, phenotypic profiles, and other molecular states. Cytoscape enabled us to construct and customize network graphs, enhancing our ability to interpret complex relationships and identify key proteins or clusters within the network that might play crucial roles in the pathogenesis of glioma influenced by BPA exposure.

### 4.5. Single-Sample Gene Set Enrichment Analysis (ssGSEA)

In our study, we utilized single-sample Gene Set Enrichment Analysis (ssGSEA) as a variant of conventional Gene Set Enrichment Analysis (GSEA). ssGSEA is designed to calculate an enrichment score for each gene set, representing the degree to which the genes in a particular set are coordinately upregulated or downregulated within a sample. This method allows for a more personalized assessment of pathway activity in individual samples, which is particularly useful in studies like ours that investigate the varying impact of environmental factors such as bisphenol A (BPA) across different samples. To conduct the ssGSEA, we first prepared an input file containing normalized gene expression data from our glioma cohort. These data provided the foundational matrix for conducting the enrichment analysis. We then selected a predefined gene list known to be associated with the bisphenol-related signature, which included genes either known or hypothesized to interact with or respond to BPA. ssGSEA then calculated an enrichment score for each sample based on the expression of this bisphenol-related gene set. The enrichment score essentially quantified the degree to which the set of interest was overrepresented at the top or bottom of the ranked list of genes in the sample, reflecting the activation state of the gene set.

### 4.6. Building a Risk Model Utilizing Genes Associated with BPA in the Glioma Population

To identify genes associated with prognosis in our glioma cohort, we initially performed a univariate Cox regression analysis. This method allowed us to evaluate the individual impact of each gene on survival time, identifying those genes with significant prognostic value. Following the identification of potential prognostic genes, we applied the Least Absolute Shrinkage and Selection Operator (LASSO) regression algorithm for dimensionality reduction. LASSO is particularly useful in reducing overfitting and handling multicollinearity by penalizing the absolute size of the regression coefficients and effectively selecting a subset of variables for the model. Upon narrowing down the significant genes through LASSO, we constructed a prognostic prediction model using multivariate Cox regression analysis. This approach helped integrate multiple genes into a single model to predict survival outcomes more accurately. Each patient was assigned a risk score based on the expression levels of the genes in the final model, reflecting their relative risk of adverse outcomes. To validate the predictive accuracy and stability of our prognostic model, we utilized receiver operating characteristic (ROC) curves. The area under the curve (AUC) of the ROC was calculated, with an AUC exceeding 0.7, indicating satisfactory predictive power. Finally, we employed Kaplan–Meier survival curves to compare the prognosis between low-risk and high-risk groups as delineated by the model.

### 4.7. Nomograms and Calibration Curves

To integrate clinical characteristics with our prognostic models, we developed a nomogram using the nomogramEx package in R software (version 4.0.3). A nomogram is a graphical representation tool that provides a statistical prognostic model to predict the probability of a clinical event, such as disease progression or survival. This tool allows for the incorporation of multiple prognostic factors, including both clinical variables and gene expression data derived from our previous analyses. The nomogram was constructed to predict individual patient outcomes based on a combination of the identified prognostic genes and relevant clinical features. Each variable in the nomogram is assigned a score, and the sum of these scores correlates with the probability of a clinical outcome. This approach enables clinicians and researchers to visualize the relative contribution of each factor to a prognosis and make individualized predictions based on a patient’s specific data. To evaluate the accuracy of our nomogram in predicting survival, we employed calibration curves. These curves compared the predicted survival probabilities against the observed outcomes. A well-calibrated model was expected to have a calibration curve that closely aligned with the 45-degree line, indicating that the predicted probabilities were close to actual outcomes. By assessing the discrepancy between actual and predicted survival with calibration curves, we were able to validate the reliability and accuracy of our nomogram as a predictive tool in clinical settings.

### 4.8. Cell Culture

Glioma cell lines (U87, U251, and Ln229) were procured from the Chinese Academy of Science. Cells were cultured within a meticulously maintained, sterile, and non-toxic milieu, wherein a consistent temperature of 37 °C was upheld. The ambient atmosphere encompassed a composition of 95% air and 5% CO_2_. The culture medium used was DMEM, which was enriched with 10% FBS (fetal bovine serum) + 1% dual antibiotic (penicillin and streptomycin, P/S) for optimal growth and maintenance. The culture medium and all supplements were purchased from Sanofi (Shanghai, China). The replacement of the growth medium occurred every other day, ensuring optimal conditions for cell growth and proliferation. We rejuvenated the cells every two months. Bisphenol A was purchased from Aladdin, CAS No.: 80-05-7, and was solubilized in DMSO to a working concentration and then acted on the cells.

### 4.9. Cell Counting Kit-8 (CCK8) Assay

Cell proliferation was evaluated using a CCK-8 assay. Overnight cultures were cultivated with three glioma cell types (U87, U251, and Ln229) seeded in 96-well plates at a density of 1 × 10^3^ cells. Once the cells reached confluence, they were treated with a medium containing various concentrations of bisphenol A for 0–96 h. The cell count was assessed at various time points using a cell counting kit (CCK-8, Shenggong, Shanghai, China). The experiment consisted of five BPA concentration gradients (0.01 µM, 0.1 µM, 1 µM, 10 µM, 100 µM), with PBS utilized as the control.

### 4.10. Transwell Assay

The upper portions of 24-well plates, previously coated with Matrigel, were inoculated with glioma cells that had been exposed to 0.1 µM BPA in a serum-free medium at a density of 2 × 10^3^ cells per well. In order to conduct control experiments, cells without BPA treatment were also included. The bottom part of the plates contained a full medium. Following a 24 h incubation period, 4% paraformaldehyde fixation and crystal violet staining were required before capturing microscopic photographs.

### 4.11. 5-Ethyl-2′-Deoxyuridine (EdU) Assay

Cell proliferation was assessed by utilizing the Cell-Light EdU DNA kit (Shenggong, Shanghai, China). The cells were meticulously cultivated in 96-well plates, employing a medium comprising 50 mM EdU at a concentration of 100 ul/well. After being incubated at 37 °C and a CO_2_ concentration of 5% for 2 h, the cells were fixed with 4% paraformaldehyde for 30 min. Then, the cells were exposed to 0.5% Triton-X-100 in PBS for a duration of 20 min. The proliferation rate was determined following the manufacturer’s provided guidelines. In order to conduct a thorough analysis, we captured images of five randomly chosen areas from each group using a fluorescence microscope.

### 4.12. Wound Healing Assay

In the wound healing assay, linear wounds were deliberately generated on three glioma cell layers that were approximately 80–90% fused utilizing a 200 μL pipette. Following that, the original medium was substituted with a serum-free medium that was enriched with BPA (0.1 μM) for subsequent incubation. The wounds were segregated into two distinct groups: one group was subjected to additional incubation with BPA (0.1 μM), while the other group was incubated without BPA. Following the designated incubation period, the wounds were meticulously examined under a microscope and comprehensive images were captured for documentation purposes.

### 4.13. Colony Formation

The effect of BPA on the proliferation of three lines of glioma cells was evaluated using a clonogenic assay. Glioma cells were seeded in 6-well plates at a density of 1000 cells per well and cultured in the presence or absence of BPA (0.1 μM). After a period of 10 days, the colonies were fixed in a solution containing 2% paraformaldehyde for a duration of 10 min. Following fixation, the colonies were stained using a solution of 0.1% crystal violet and subsequently captured in photographs for quantification. To ensure reliability, all experiments were independently repeated on three separate occasions.

### 4.14. Flow Cytometry Analysis of Apoptosis

We utilized the Annexin V-Alexa Fluor 647/PI Apoptosis Detection Kit (manufactured by Yeasen Biotechnology in Shanghai, China) according to the provided protocol to evaluate the initiation of apoptosis in glioma cells by BPA. Specifically, Ln229, U251, and U87 glioma cells were exposed to a concentration of 0.1 µM BPA for a duration of 48 h. Subsequently, the collected cells were double-stained with Annexin V-FITC/PI and analyzed using the CytoFLEX S Flow Cytometer (manufactured by Beckman Dickinson in the 250 South Kraemer Boulevard, Brea, CA 92821, USA). To evaluate the rate of cell apoptosis, the FlowJo (version 10.0) was employed.

### 4.15. High-Content Screening Digital Photon Counting (DPC)

The experiment incorporated three glioma cell lines that were devoid of fluorescence labeling. The live cells were subsequently subjected to real-time imaging, employing digital photon counting (DPC) mode imaging within the Operetta CLS High-Intensity Screening System. This approach facilitated the dynamic observation of cellular activity and proliferation. To support cell growth, a 96-well, glass-bottomed, high-content screening microtiter plate was employed, with each well containing 1500 cells and a pre-mixed medium of 0.1 µM BPA. The cells were then captured with real-time images at 12 h intervals, commencing from 0 h, with cell counting being automatically performed by the high-content screening system.

### 4.16. Statistical Analysis

The outcome graphs derived from EdU assays were analyzed using ImageJ software(Version:2.1.0) in order to interpret the number of positive cells for result enumeration. The cell migration areas in the wound healing assays were determined by employing ImageJ software. Similarly, the result graphs of the colony formation experiments were established in ImageJ software to ascertain the sizes of the colonies. Furthermore, the invasive and migrating cells in Transwell experiments were also quantified using ImageJ software. Lastly, the counts of viable cells at various time points in the DPC experiments were automatically generated by the high-content imaging system Operetta CLS. A *p*-value below 0.05 was deemed to indicate statistical significance for all statistical analyses conducted using R Software (version 4.0.3). All bioinformatics analyses were performed on a high-performance computing platform to ensure the efficient processing of large datasets. The choice of software, versions, and specific parameters used for each analysis step is documented in the Supplementary Materials, ensuring the reproducibility and transparency of the bioinformatics workflows.

## 5. Conclusions

In summary, our study elucidates the association between BPA and gliomas, demonstrating that the effect on glioma proliferation is dependent on both the dose and duration of exposure. BPA has the potential to modulate crucial cellular mechanisms such as cell proliferation and migration and the inhibition of apoptosis by activating ER receptors. We developed a nomogram that incorporates gender, tumor grade, and age in order to enhance the predictive capacity for glioma, ultimately aiming to improve predictive accuracy. Furthermore, BPA enhances glioma invasion and migration and may augment the EMT characteristics of glioma cells while also promoting cell viability and reducing apoptosis. These findings shed light on the impact of bisphenol-related compounds, specifically BPA, on gliomas. While further research is imperative, these results provide valuable insights into the potential effects of BPA on the development and progression of gliomas.

## Data Availability

The data presented in this study are available on request from the corresponding author.

## Supplementary Materials

The following supporting information can be downloaded at: https://www.mdpi.com/article/10.3390/ijms25052504/s1.
