# Supplementary material for: Bisphenol A: Unveiling Its Role in Glioma Progression and Tumor Growth
Source: Int J Mol Sci. 2024 Feb 21;25(5):2504. doi: 10.3390/ijms25052504 (PMC10931199; doi:10.3390/ijms25052504)

	pvalue	Hazard ratio
DSG2	<0.001	1.505(1.314−1.724)
CP	<0.001	1.304(1.176−1.445)
CHI3L2	<0.001	1.332(1.227−1.447)
LYZ	<0.001	1.354(1.214−1.510)
SRPX2	<0.001	1.667(1.475−1.885)
MET	<0.001	1.334(1.189−1.496)
HOXA2	<0.001	1.647(1.476−1.838)
ABCC3	<0.001	1.499(1.377−1.632)
CD69	<0.001	1.482(1.276−1.721)
CD2	<0.001	1.682(1.449−1.952)
GBP1	<0.001	1.530(1.377−1.699)
PLAU	<0.001	1.472(1.326−1.635)
LIF	<0.001	1.421(1.277−1.582)
CHI3L1	<0.001	1.336(1.262−1.414)
POSTN	<0.001	1.405(1.306−1.512)
SPOCD1	<0.001	1.340(1.225−1.467)
ANXA1	<0.001	1.467(1.345−1.600)
OASL	<0.001	1.531(1.356−1.729)
WDR38	<0.001	1.368(1.218−1.537)
CXCL9	<0.001	1.421(1.267−1.593)
GPR65	<0.001	1.581(1.357−1.842)
TRPM8	<0.001	1.640(1.444−1.862)
COL8A1	<0.001	1.720(1.547−1.911)
TCTEX1D1	<0.001	1.436(1.297−1.590)
GBP5	<0.001	1.692(1.458−1.962)
HPD	<0.001	1.656(1.416−1.938)
CLIC6	<0.001	1.640(1.373−1.960)
CCR5	<0.001	1.611(1.378−1.883)
ZNF474	<0.001	2.007(1.683−2.392)
C7orf57	<0.001	1.379(1.246−1.527)
CA3	<0.001	1.522(1.389−1.667)
OSR2	<0.001	1.489(1.305−1.700)
NNMT	<0.001	1.409(1.302−1.525)
IL7R	<0.001	1.779(1.484−2.133)
CXCL10	<0.001	1.374(1.261−1.498)
CXCL11	<0.001	1.583(1.403−1.787)
SAA1	<0.001	1.432(1.320−1.552)
CHRNA9	<0.001	1.507(1.345−1.689)
HOXC10	<0.001	1.553(1.401−1.720)
SOCS3	<0.001	1.322(1.209−1.446)
FAM183A	<0.001	1.407(1.281−1.546)
PLA2G2A	<0.001	1.366(1.248−1.496)
HLA−DQA1	<0.001	1.303(1.180−1.440)
RASSF9	<0.001	1.423(1.266−1.599)
FCGR3A	<0.001	1.305(1.174−1.451)
IGLV7−46	<0.001	1.397(1.227−1.590)
CFAP45	<0.001	1.628(1.453−1.825)
HSPA7	<0.001	1.469(1.315−1.642)
GBP1P1	<0.001	1.716(1.504−1.958)
HLA−DQB2	<0.001	1.318(1.182−1.470)
FCGR2C	<0.001	1.823(1.570−2.116)
AC106865.1	<0.001	1.621(1.358−1.936)
AL354919.2	<0.001	1.627(1.396−1.896)
HP	<0.001	1.646(1.449−1.870)
CLEC5A	<0.001	1.749(1.527−2.004)
AC008760.2	<0.001	1.494(1.313−1.701)

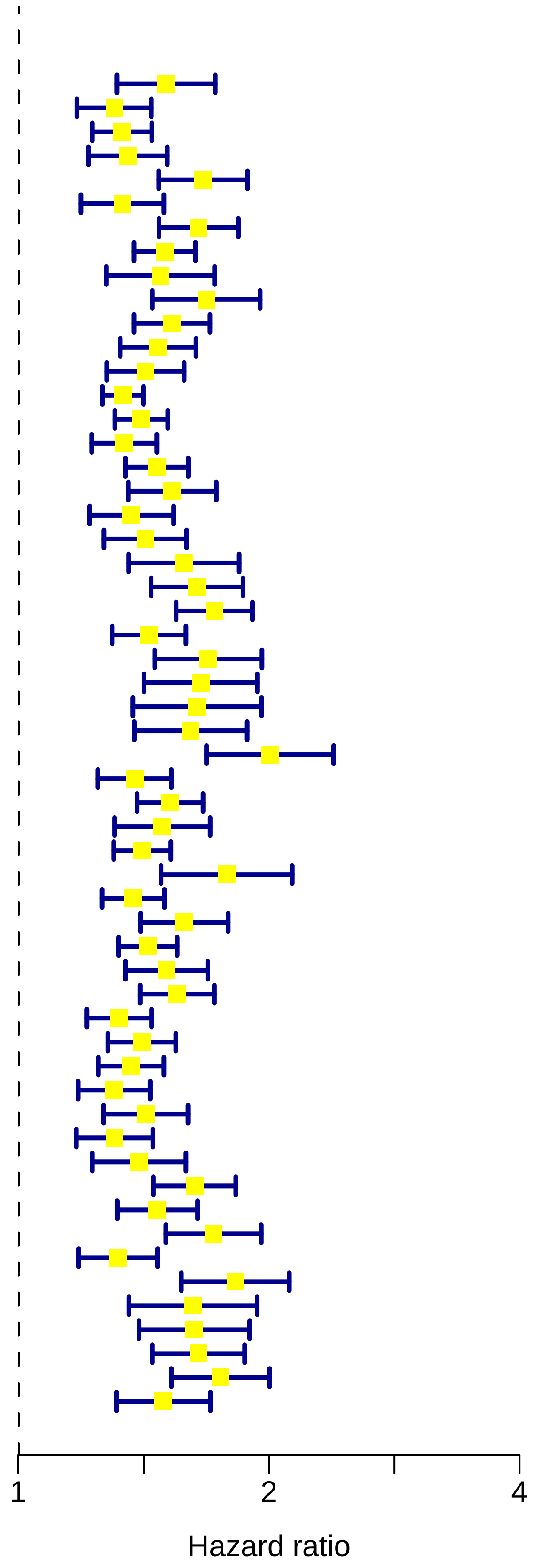

Supplement: Supplementary file 1 [file ijms-25-02504-s001.zip › ijms-2766183-supplementary/risk model/forest.pdf]
