# Supplementary figures and images for: Bisphenol A: Unveiling Its Role in Glioma Progression and Tumor Growth
Source: Int J Mol Sci. 2024 Feb 21;25(5):2504. doi: 10.3390/ijms25052504 (PMC10931199; doi:10.3390/ijms25052504)

Percent weight

100  
75  
50  
25  
0

low

high

riskScore

95%

5%

91%

9%

age

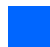

<65

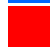

>=65

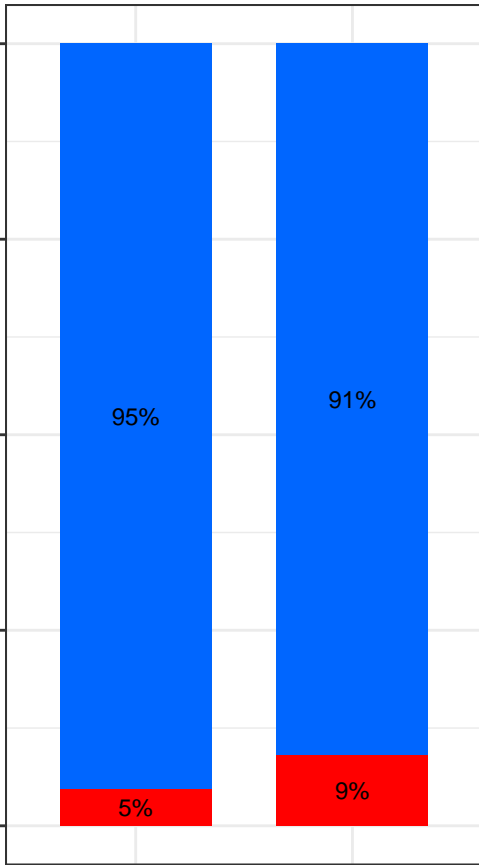

Supplement: Supplementary file 1 [file ijms-25-02504-s001.zip › ijms-2766183-supplementary/after risk model/age.barplot.pdf]

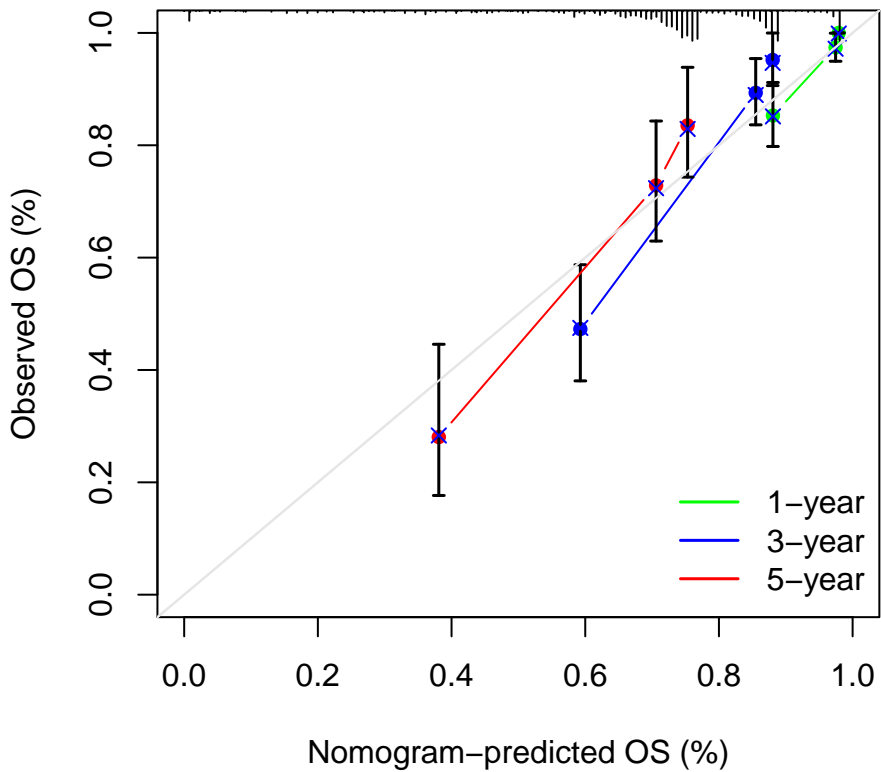

Supplement: Supplementary file 1 [file ijms-25-02504-s001.zip › ijms-2766183-supplementary/after risk model/calibration.pdf]

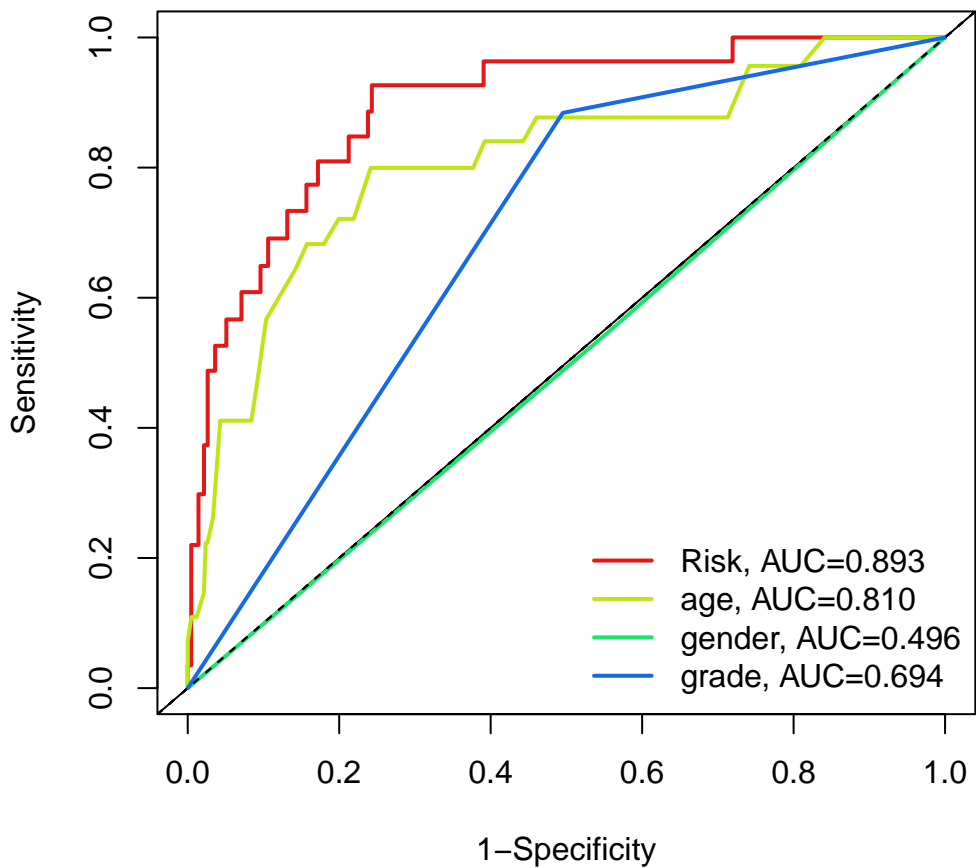

Supplement: Supplementary file 1 [file ijms-25-02504-s001.zip › ijms-2766183-supplementary/after risk model/cliROC.pdf]

Percent weight

100  
75  
50  
25  
0

low

high

riskScore

43%

46%

57%

54%

gender

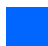

FEMALE

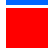

MALE

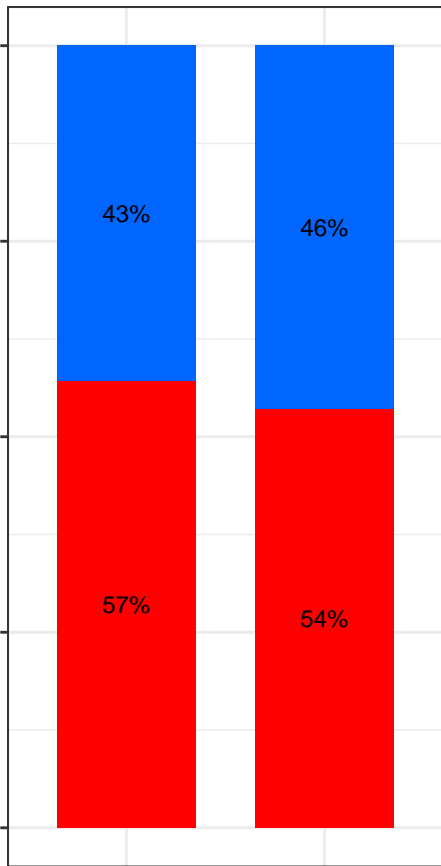

Supplement: Supplementary file 1 [file ijms-25-02504-s001.zip › ijms-2766183-supplementary/after risk model/gender.barplot.pdf]

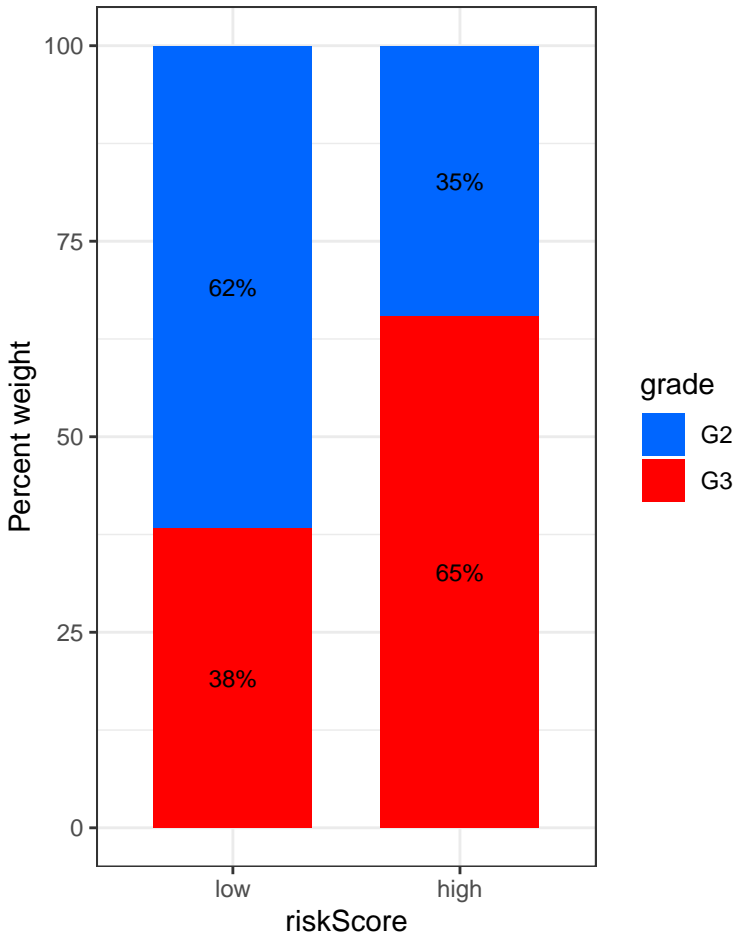

Supplement: Supplementary file 1 [file ijms-25-02504-s001.zip › ijms-2766183-supplementary/after risk model/grade.barplot.pdf]

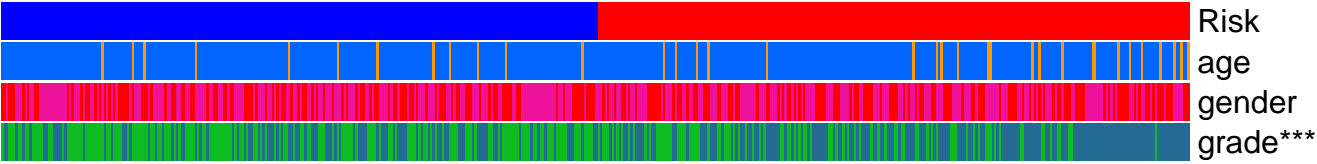

| Risk | age  | gender | grade*** |
|------|------|--------|----------|
| high | <65  | FEMALE | G2       |
| low  | >=65 | MALE   | G3       |

Supplement: Supplementary file 1 [file ijms-25-02504-s001.zip › ijms-2766183-supplementary/after risk model/heatmap.pdf]

|           | pvalue | Hazard ratio       |
|-----------|--------|--------------------|
| age       | <0.001 | 1.046(1.030–1.063) |
| gender    | 0.119  | 1.348(0.926–1.963) |
| grade     | <0.001 | 2.311(1.525–3.503) |
| riskScore | <0.001 | 1.140(1.104–1.178) |

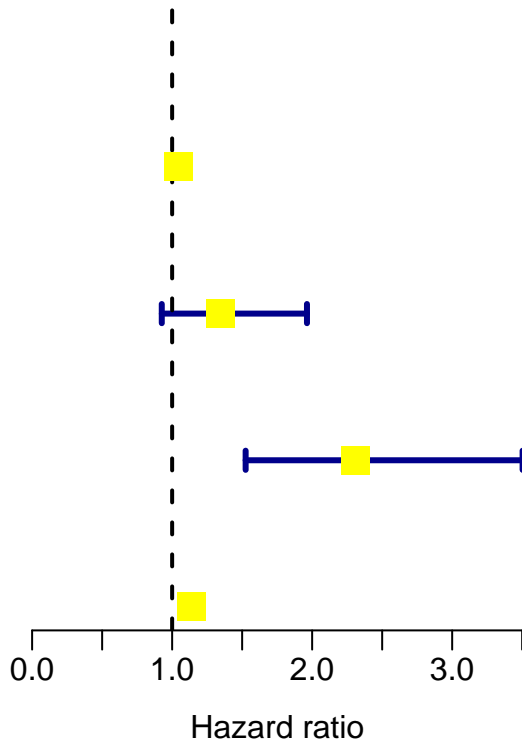

Supplement: Supplementary file 1 [file ijms-25-02504-s001.zip › ijms-2766183-supplementary/after risk model/multiForest.pdf]

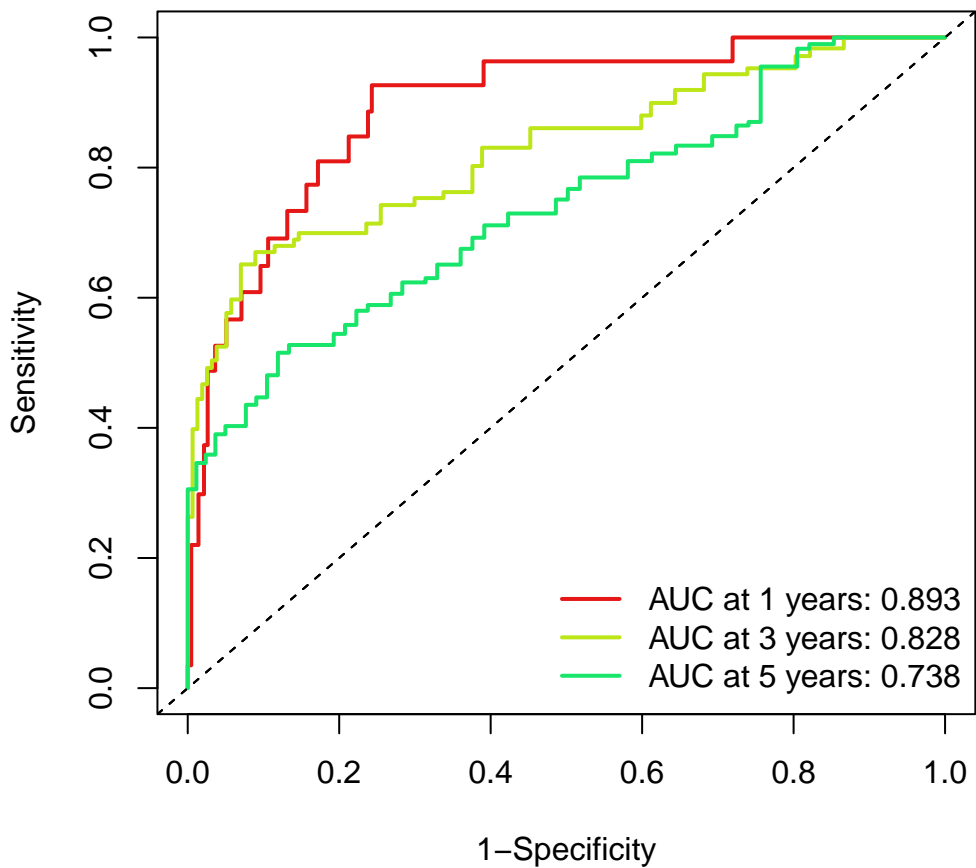

Supplement: Supplementary file 1 [file ijms-25-02504-s001.zip › ijms-2766183-supplementary/after risk model/ROC.pdf]

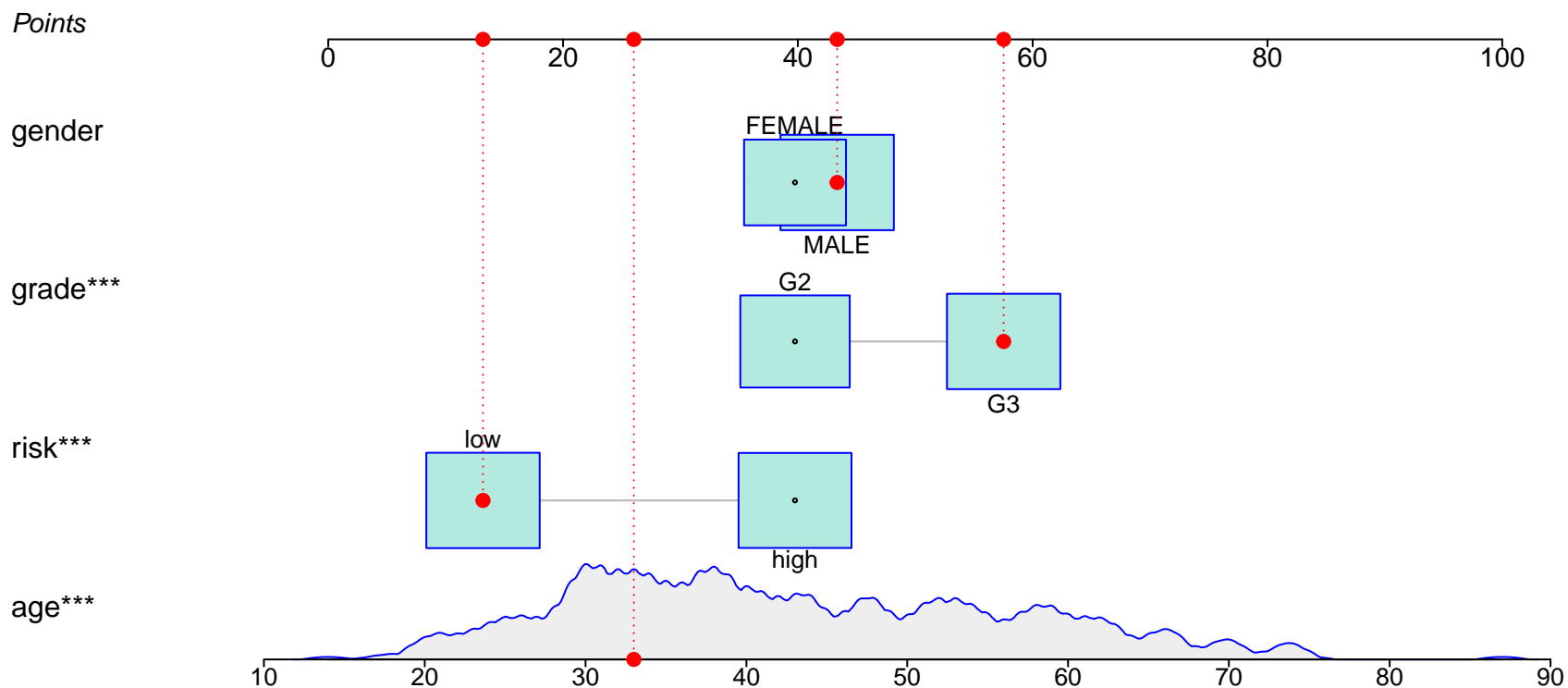

**Total points**

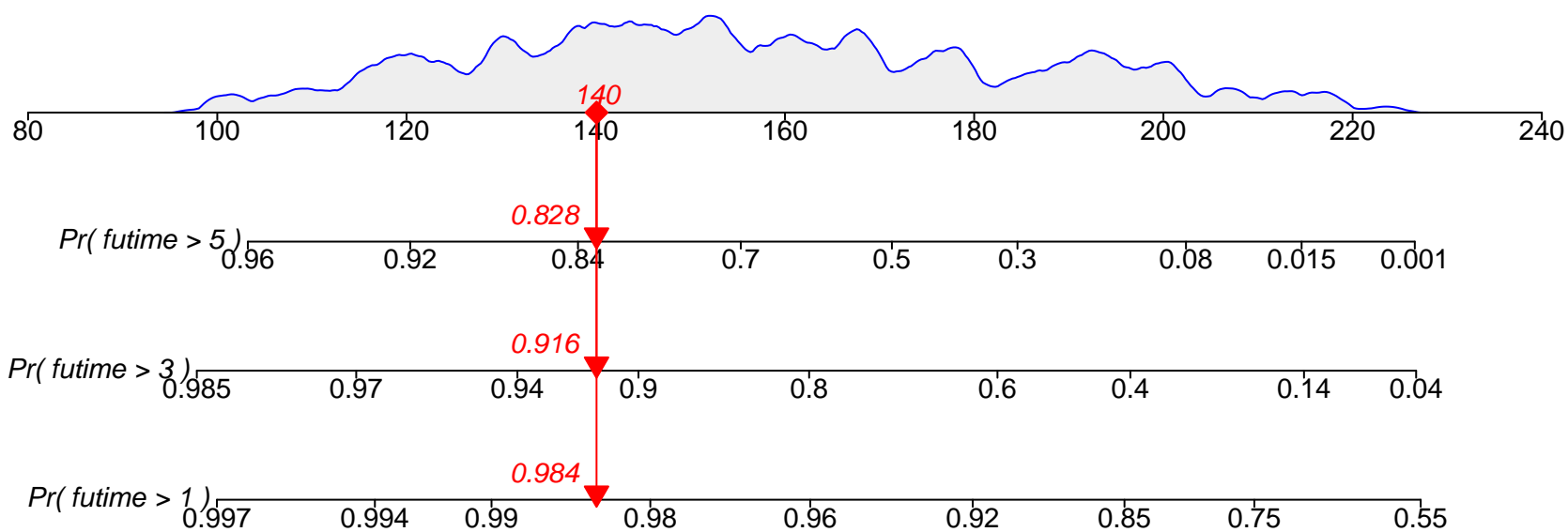

Supplement: Supplementary file 1 [file ijms-25-02504-s001.zip › ijms-2766183-supplementary/after risk model/Rplot.pdf]

HOXA2 high low

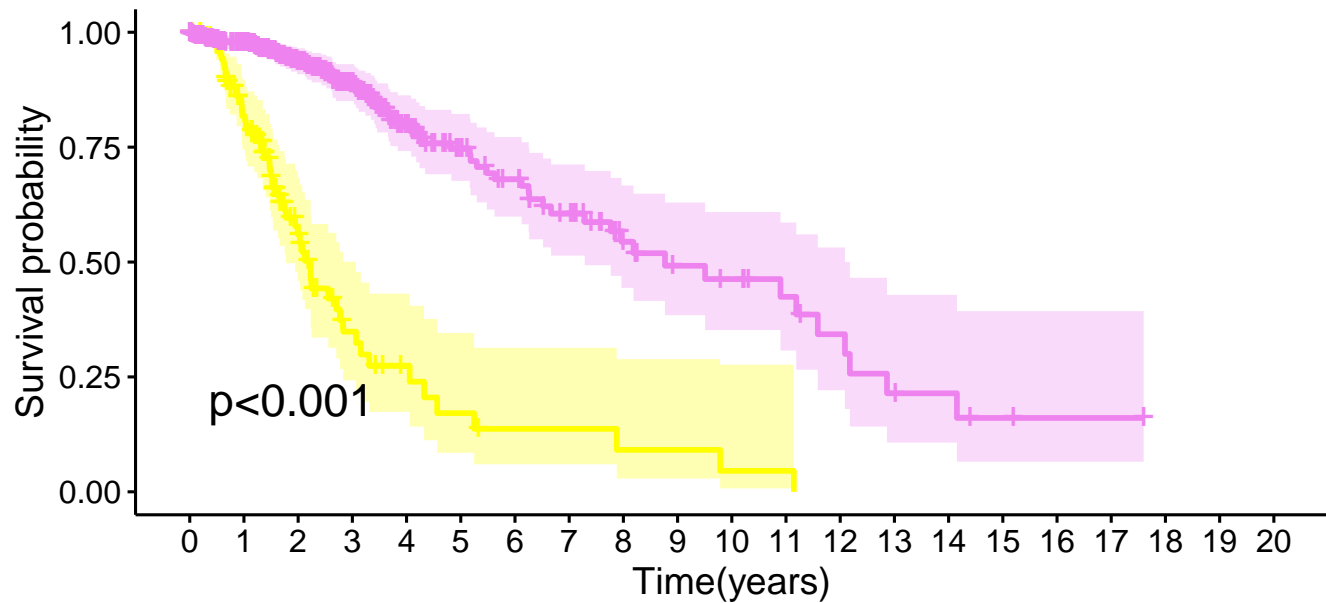

HOXA2

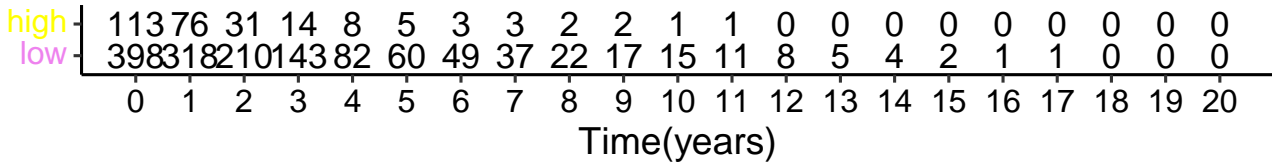

Supplement: Supplementary file 1 [file ijms-25-02504-s001.zip › ijms-2766183-supplementary/after risk model/Survival.HOXA2.pdf]

OASL    high    low

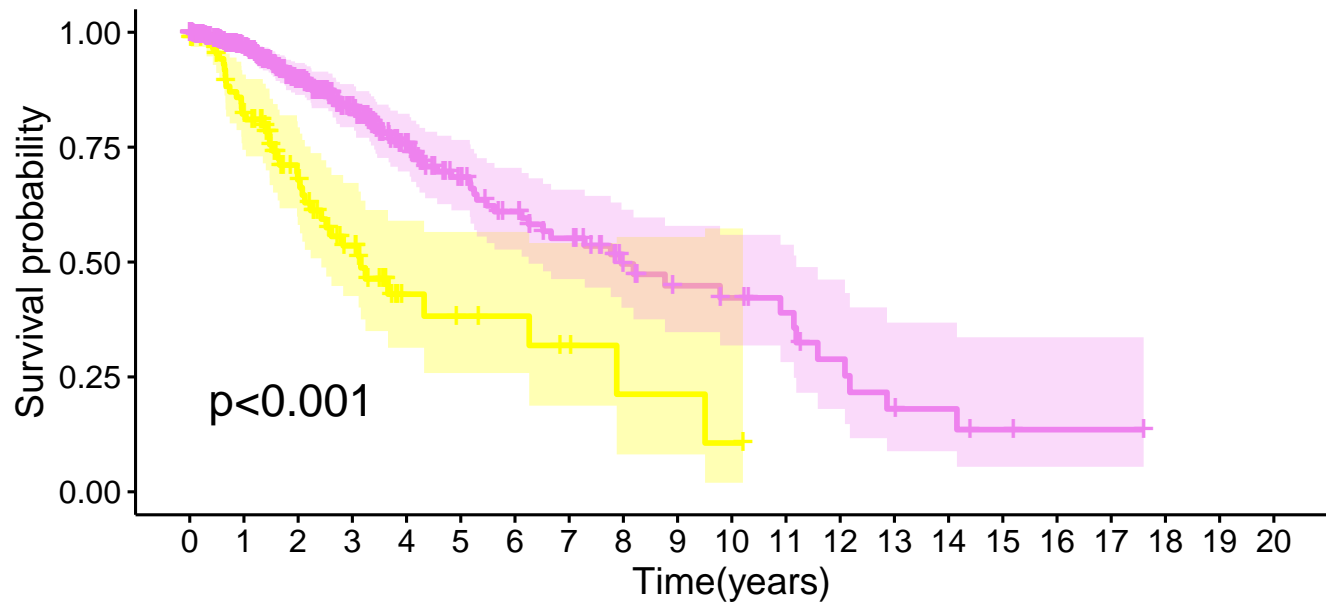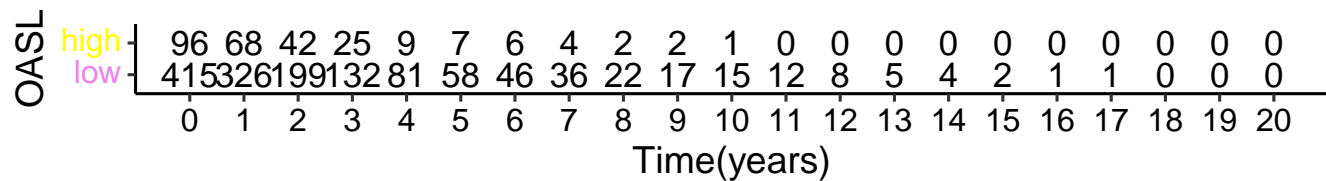

Supplement: Supplementary file 1 [file ijms-25-02504-s001.zip › ijms-2766183-supplementary/after risk model/Survival.OASL.pdf]

POSTN + high + low

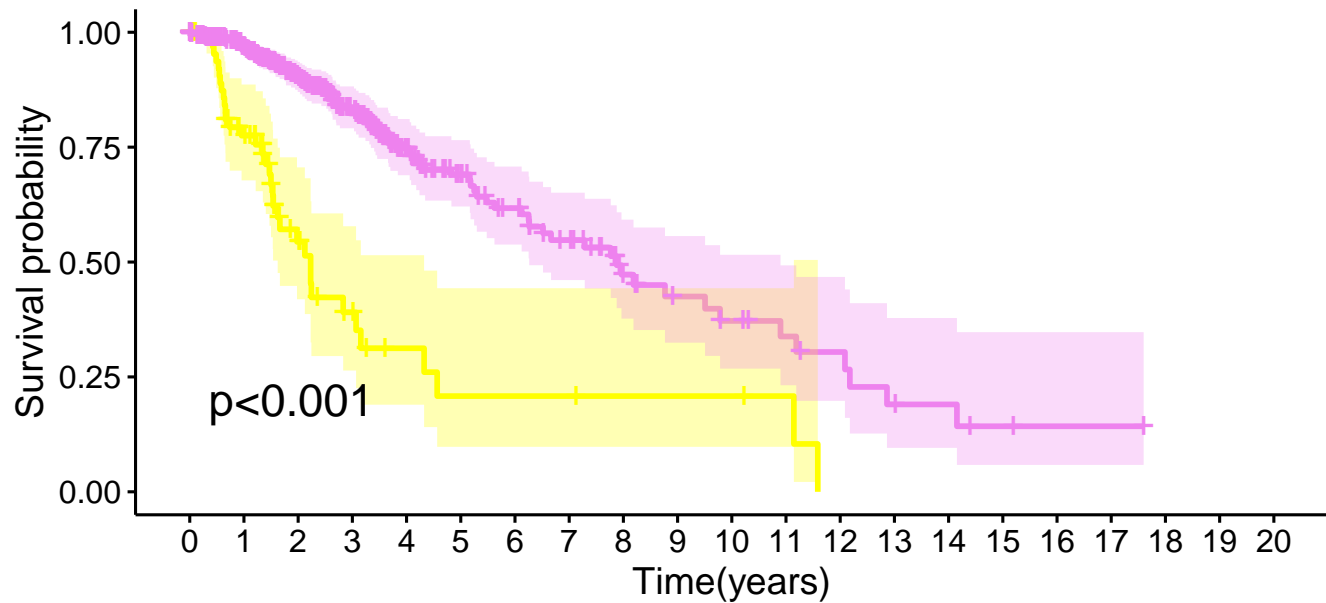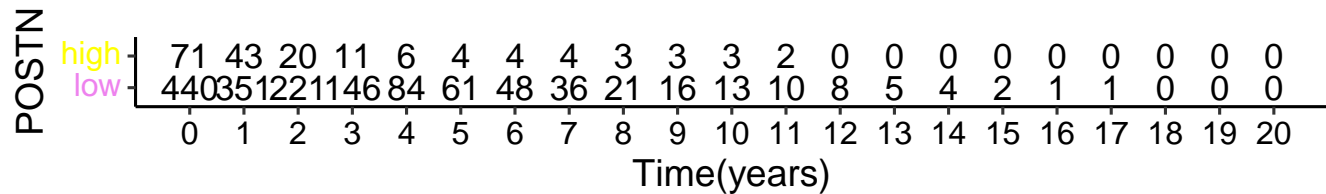

Supplement: Supplementary file 1 [file ijms-25-02504-s001.zip › ijms-2766183-supplementary/after risk model/Survival.POSTN.pdf]

ZNF474 + high + low

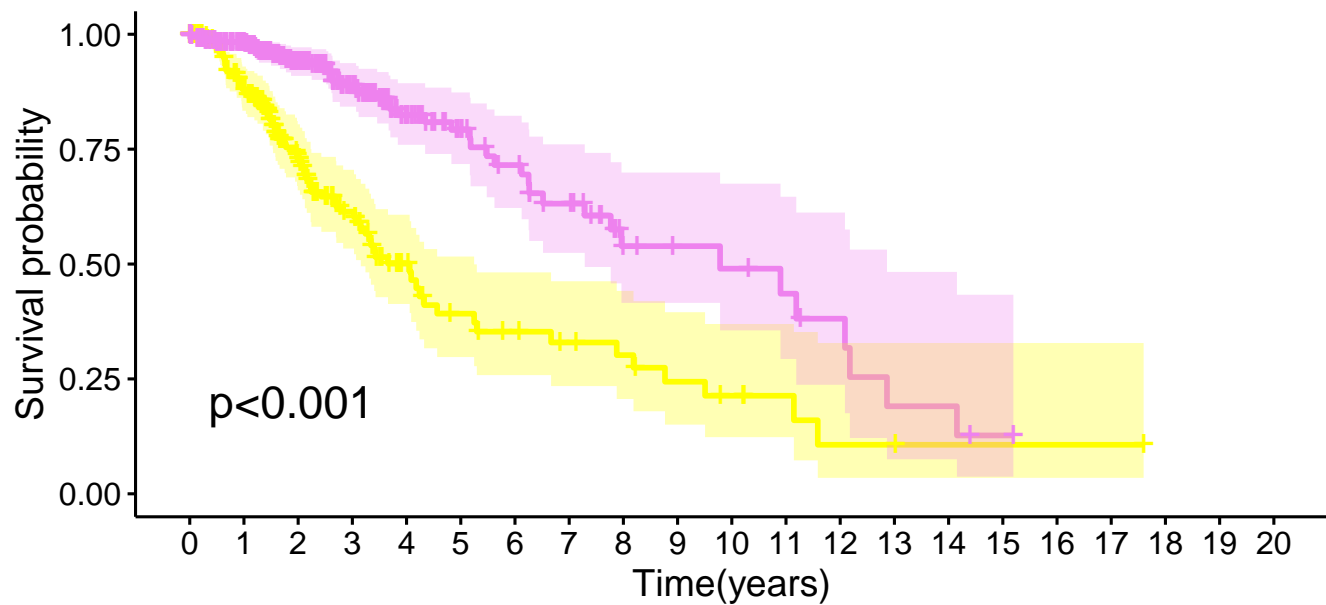

ZNF474

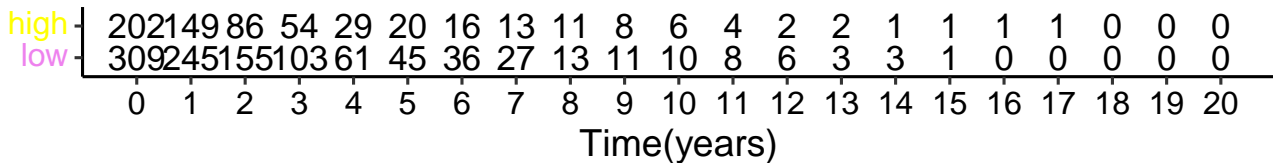

Supplement: Supplementary file 1 [file ijms-25-02504-s001.zip › ijms-2766183-supplementary/after risk model/Survival.ZNF474.pdf]

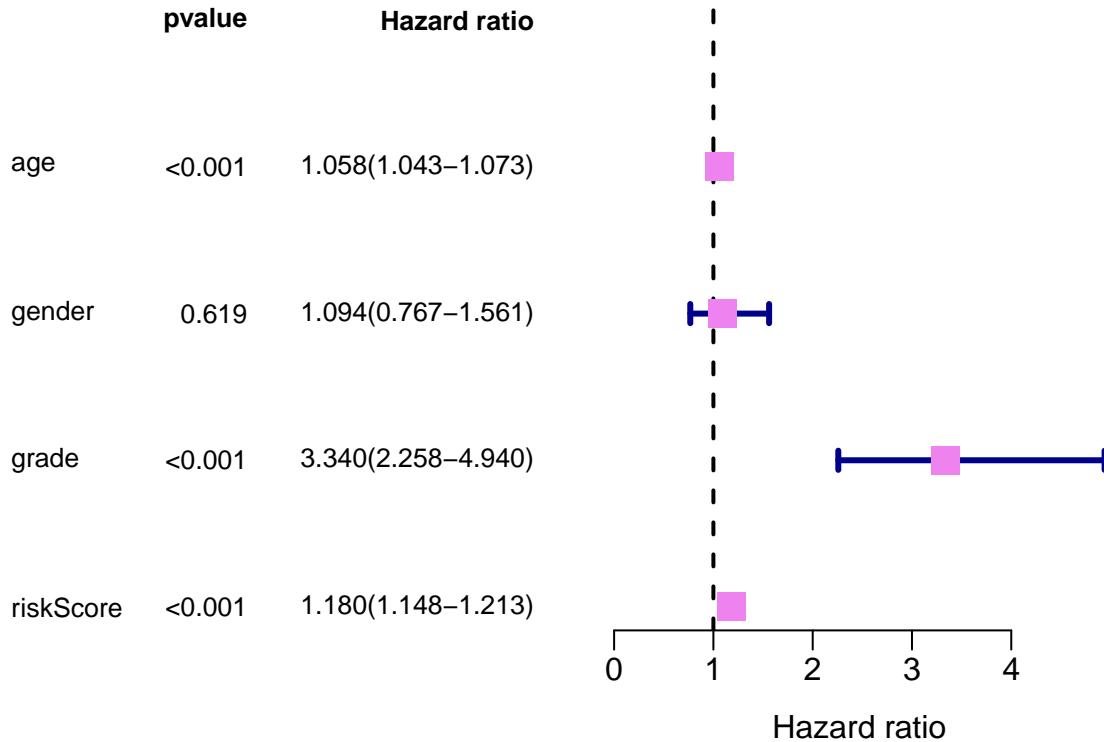

Supplement: Supplementary file 1 [file ijms-25-02504-s001.zip › ijms-2766183-supplementary/after risk model/uniForest.pdf]

Percent weight

100  
75  
50  
25  
0

low

high

Score

93%

93%

7%

7%

age

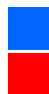

<65

>=65

Supplement: Supplementary file 1 [file ijms-25-02504-s001.zip › ijms-2766183-supplementary/clinical relation/age.barplot.pdf]

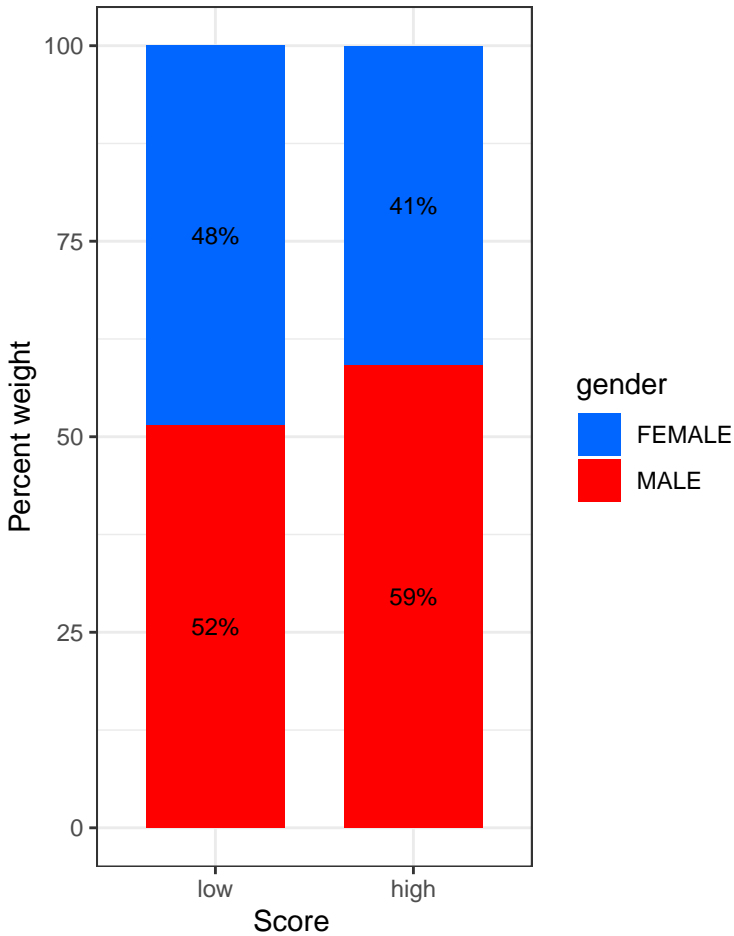

Supplement: Supplementary file 1 [file ijms-25-02504-s001.zip › ijms-2766183-supplementary/clinical relation/gender.barplot.pdf]

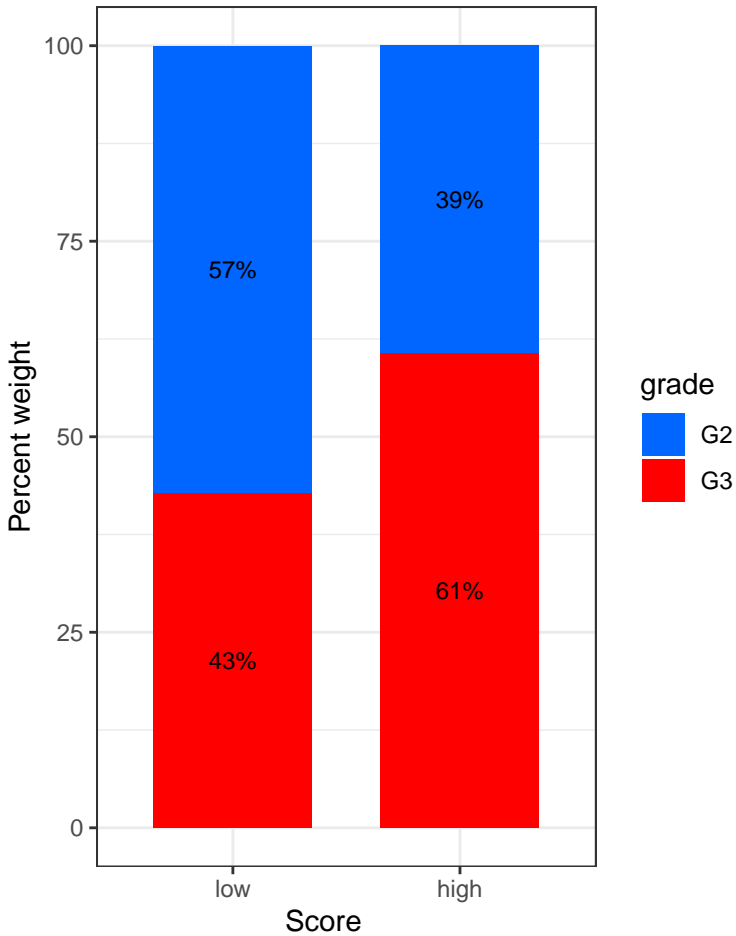

Supplement: Supplementary file 1 [file ijms-25-02504-s001.zip › ijms-2766183-supplementary/clinical relation/grade.barplot.pdf]

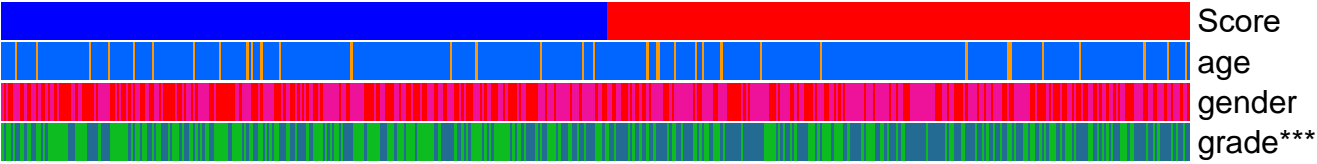

| Score | age  | gender | grade*** |
|-------|------|--------|----------|
| high  | <65  | FEMALE | G2       |
| low   | >=65 | MALE   | G3       |

Supplement: Supplementary file 1 [file ijms-25-02504-s001.zip › ijms-2766183-supplementary/clinical relation/heatmap.pdf]

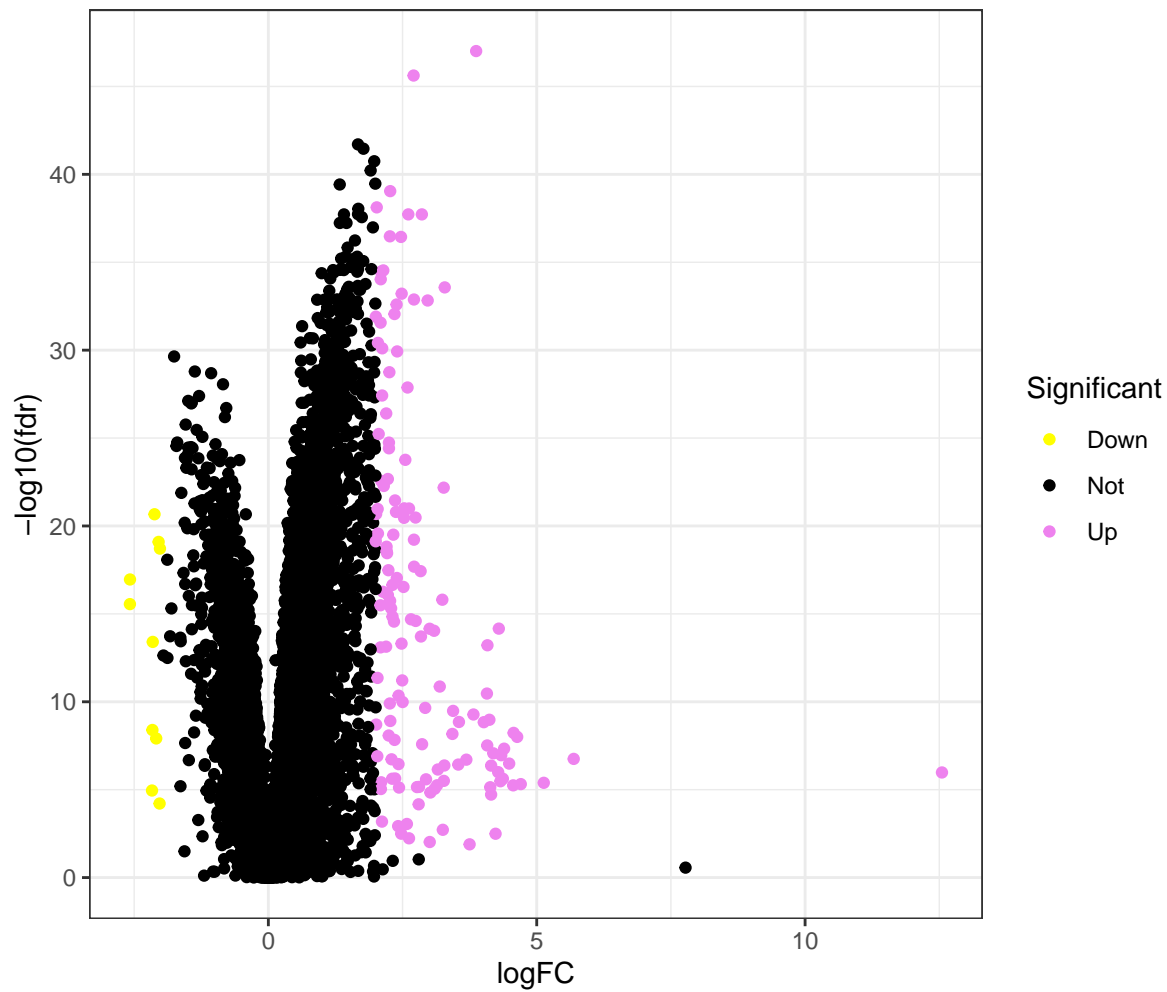

Supplement: Supplementary file 1 [file ijms-25-02504-s001.zip › ijms-2766183-supplementary/differentially expressed analysis/vol.pdf]

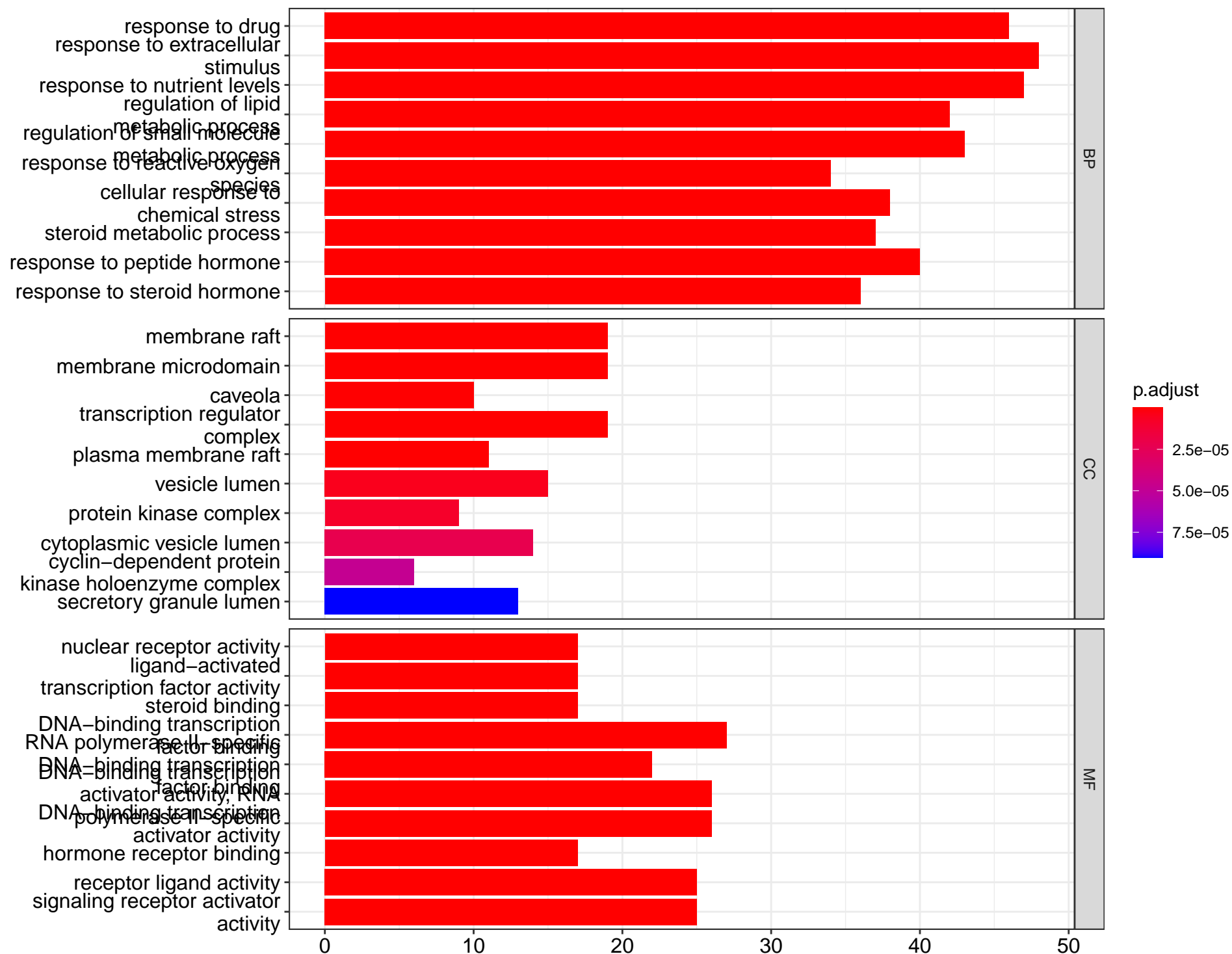

Supplement: Supplementary file 1 [file ijms-25-02504-s001.zip › ijms-2766183-supplementary/GOKEGG/barplot.pdf]

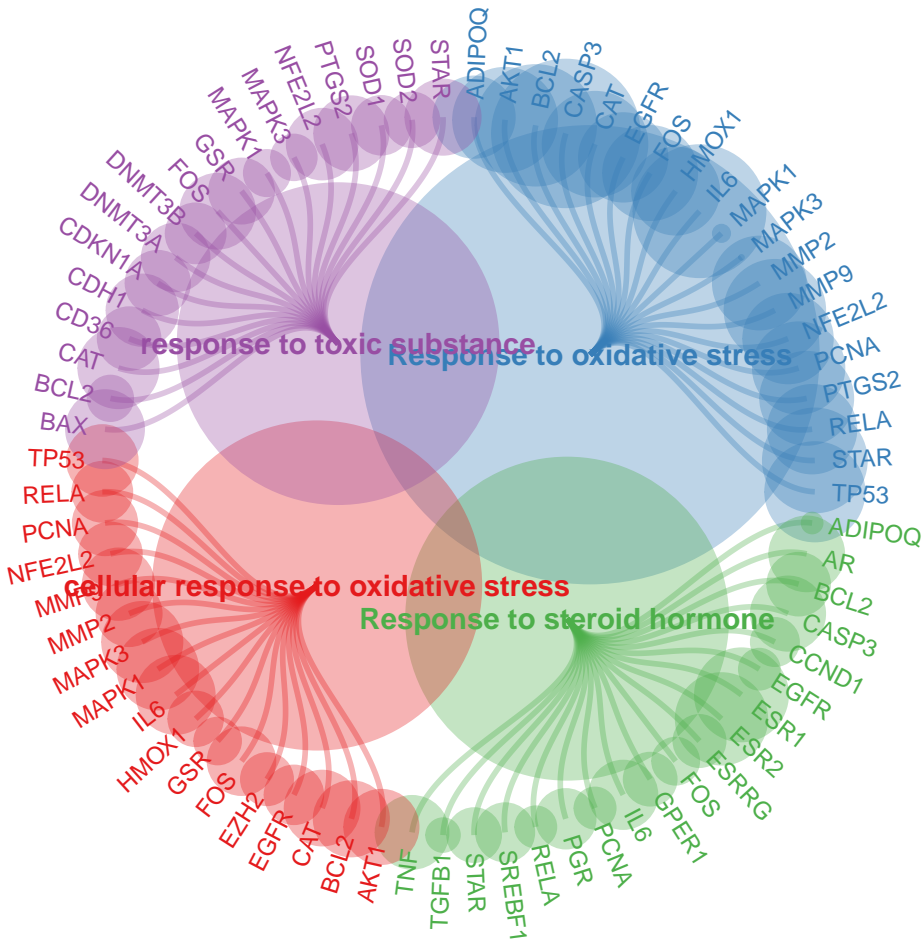

Supplement: Supplementary file 1 [file ijms-25-02504-s001.zip › ijms-2766183-supplementary/GOKEGG/BP.pdf]

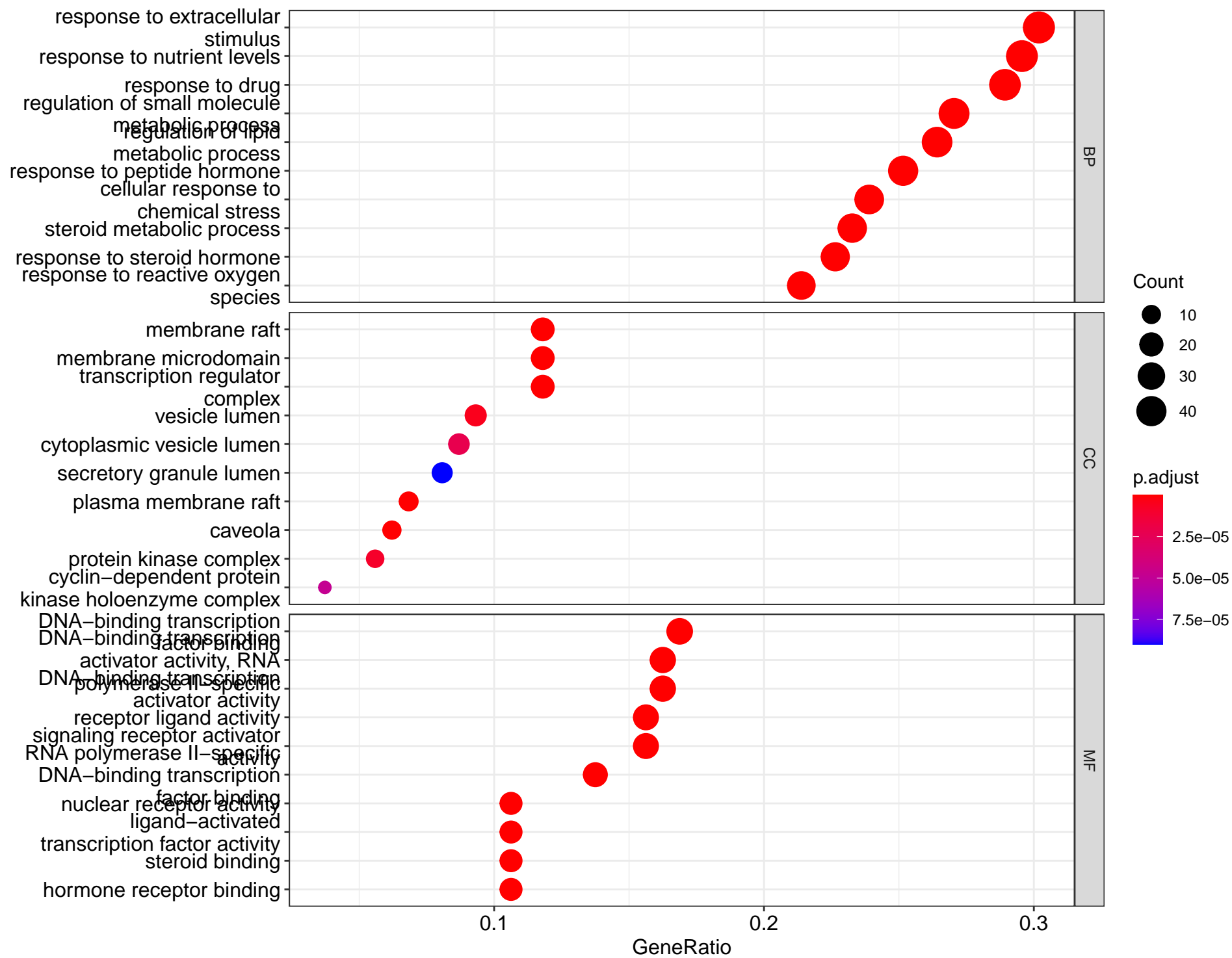

Supplement: Supplementary file 1 [file ijms-25-02504-s001.zip › ijms-2766183-supplementary/GOKEGG/bubble.pdf]

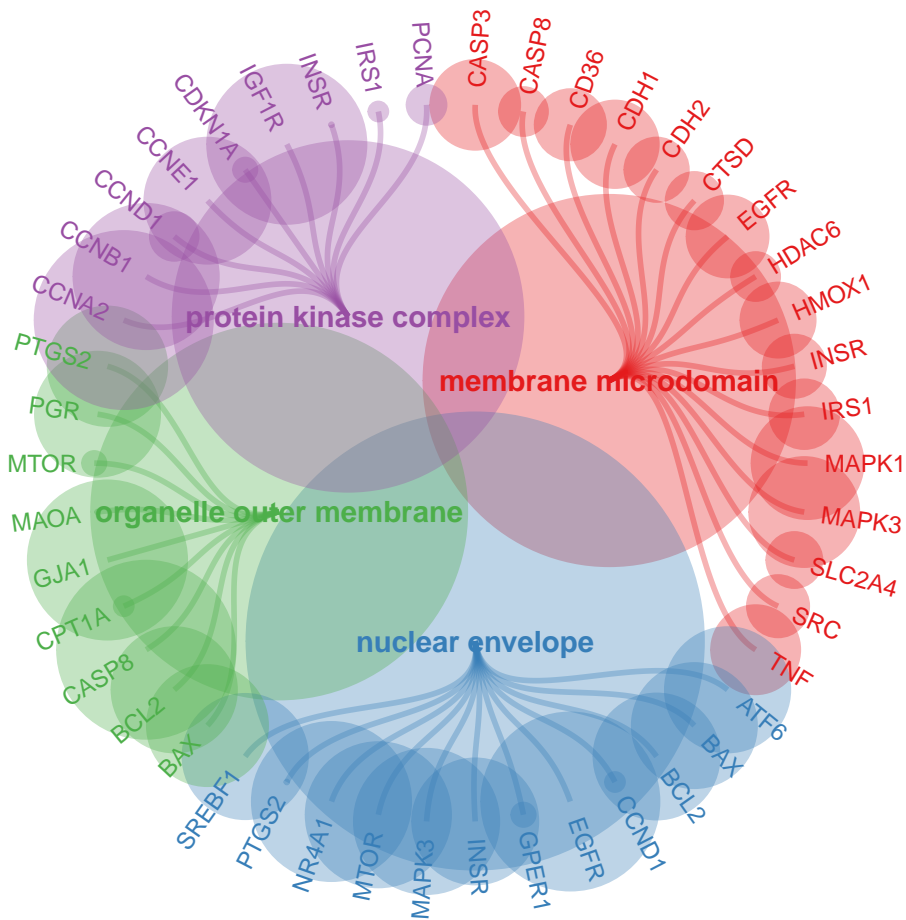

Supplement: Supplementary file 1 [file ijms-25-02504-s001.zip › ijms-2766183-supplementary/GOKEGG/CC.pdf]

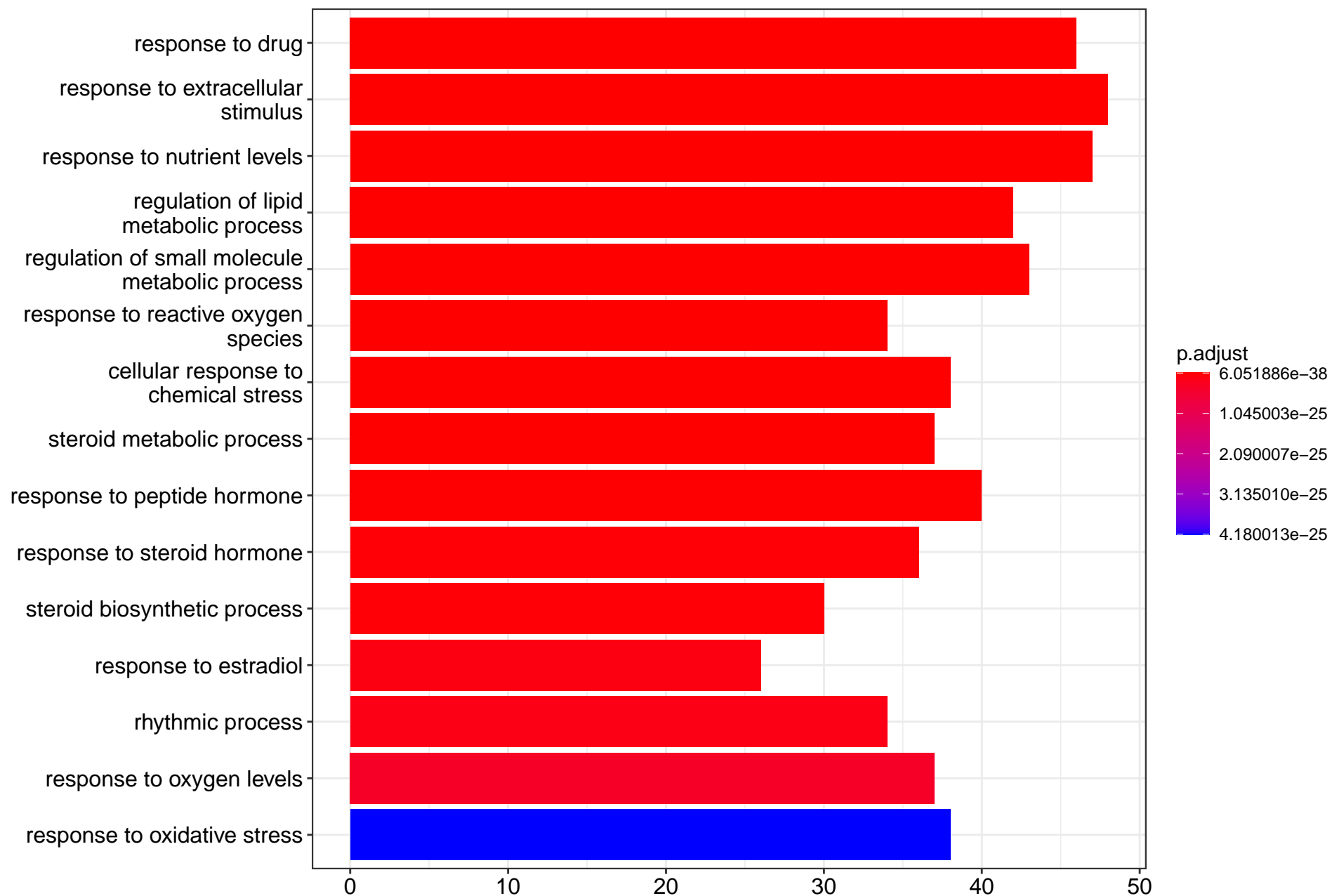

Supplement: Supplementary file 1 [file ijms-25-02504-s001.zip › ijms-2766183-supplementary/GOKEGG/KEGGbarplot.pdf]

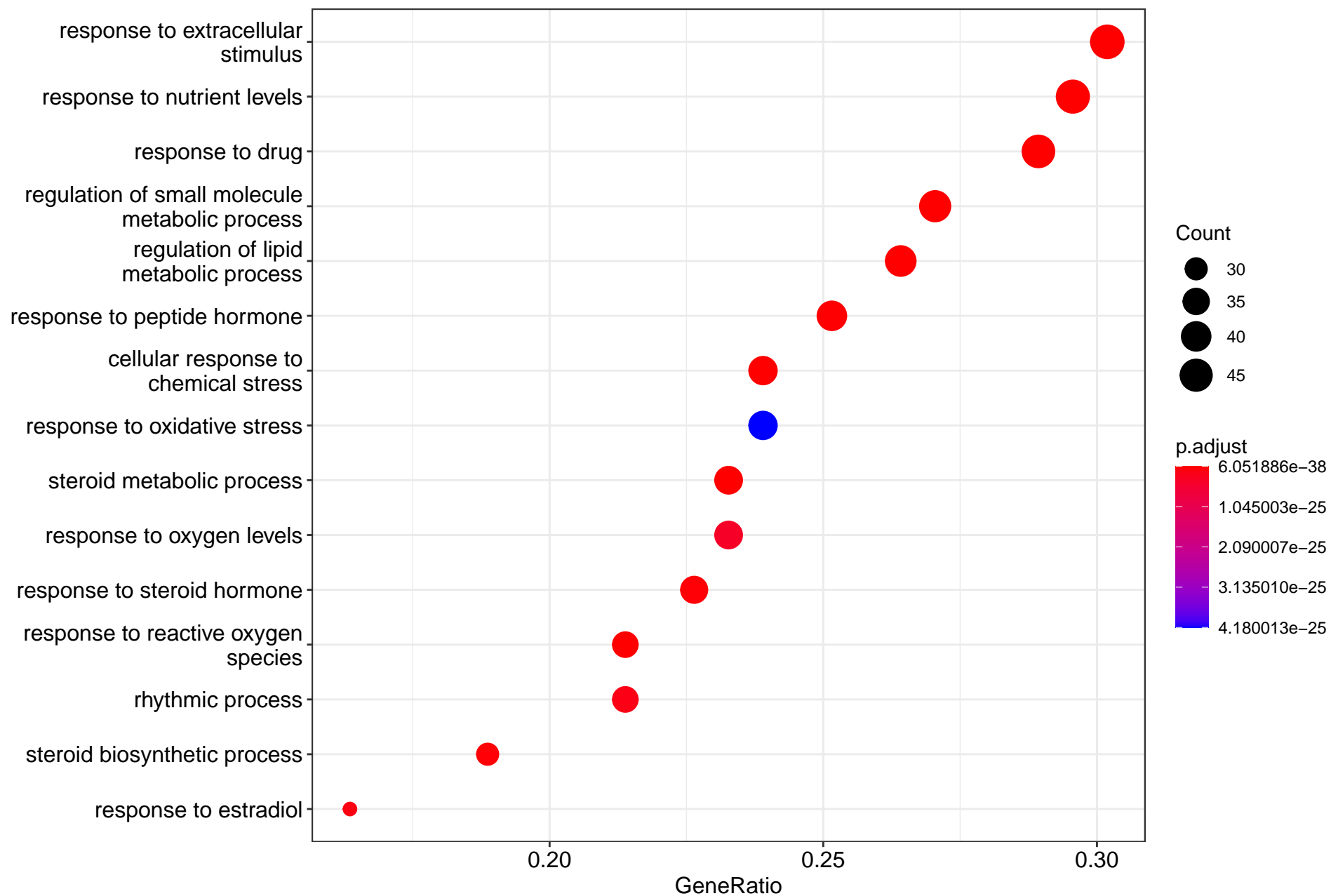

Supplement: Supplementary file 1 [file ijms-25-02504-s001.zip › ijms-2766183-supplementary/GOKEGG/KEGGbubble.pdf]

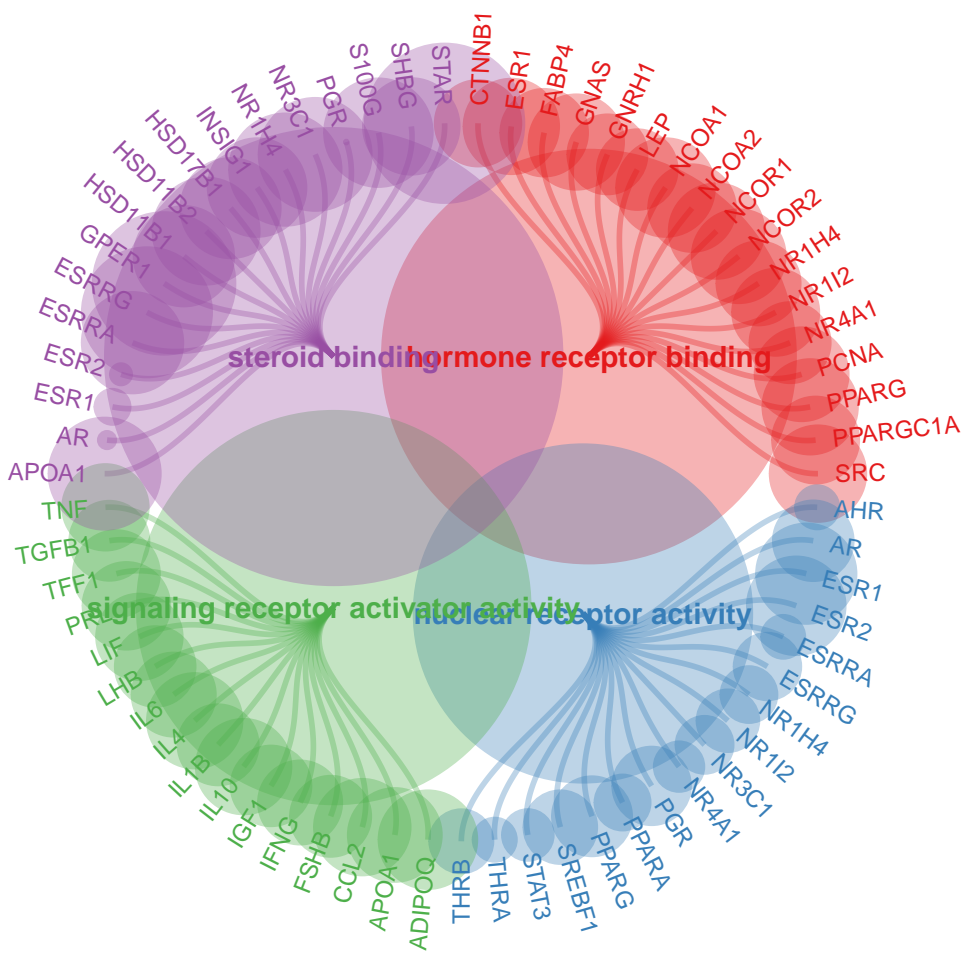

Supplement: Supplementary file 1 [file ijms-25-02504-s001.zip › ijms-2766183-supplementary/GOKEGG/MF.pdf]

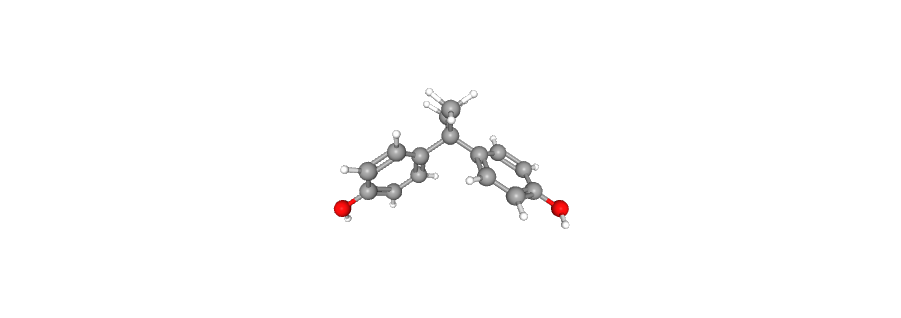

Supplement: Supplementary file 1 [file ijms-25-02504-s001.zip › ijms-2766183-supplementary/molecular docking/Bisphenol-A_Conformer3D_large (1).png]

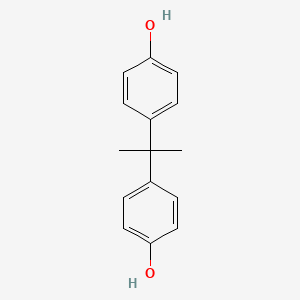

Supplement: Supplementary file 1 [file ijms-25-02504-s001.zip › ijms-2766183-supplementary/molecular docking/BPA.png]

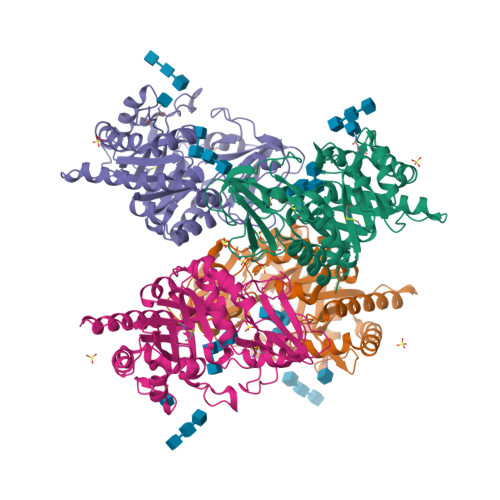

Supplement: Supplementary file 1 [file ijms-25-02504-s001.zip › ijms-2766183-supplementary/molecular docking/CHI3L1.1.jpeg]

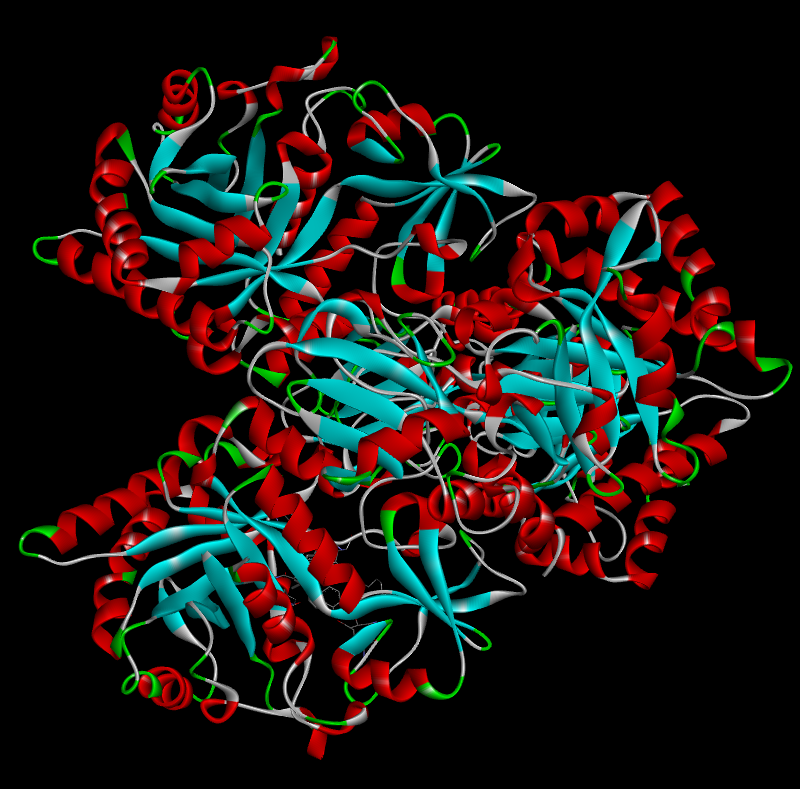

Supplement: Supplementary file 1 [file ijms-25-02504-s001.zip › ijms-2766183-supplementary/molecular docking/CHI3L1/242e41f0ead8c8500cfafd150fd4ed7.png]

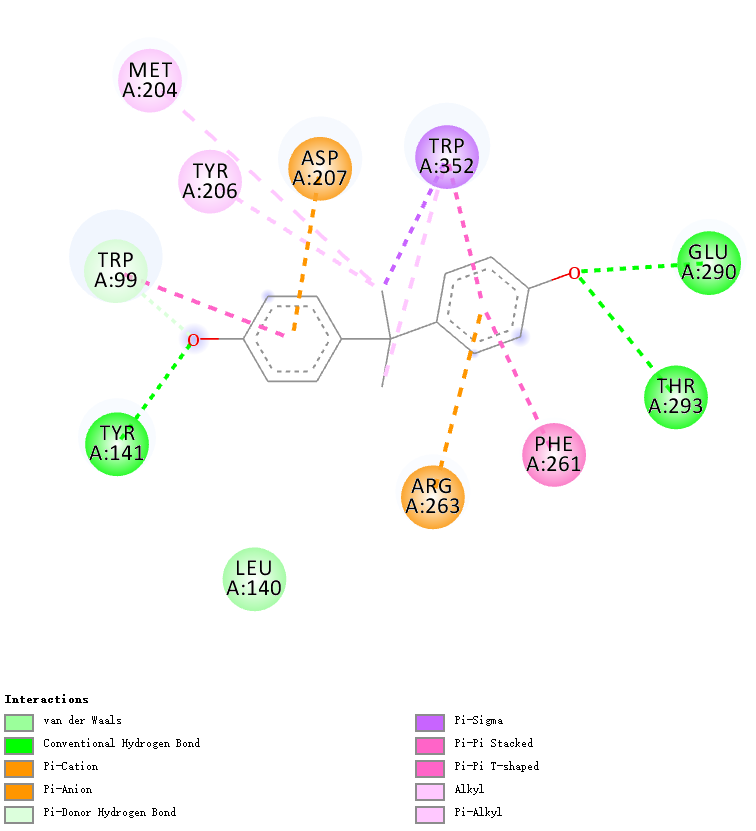

Supplement: Supplementary file 1 [file ijms-25-02504-s001.zip › ijms-2766183-supplementary/molecular docking/CHI3L1/ba8b444aaf2224a9fa44b1be9e57572.png]

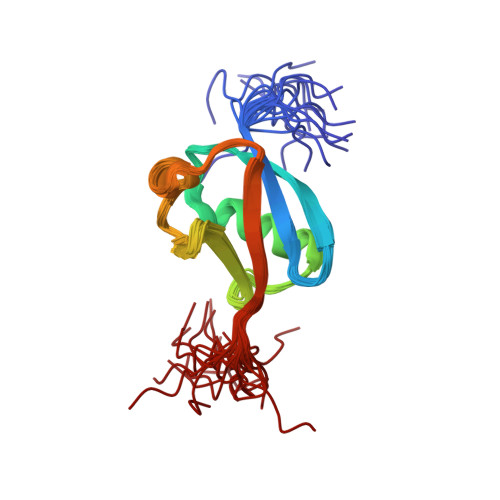

Supplement: Supplementary file 1 [file ijms-25-02504-s001.zip › ijms-2766183-supplementary/molecular docking/OASL.1.jpeg]

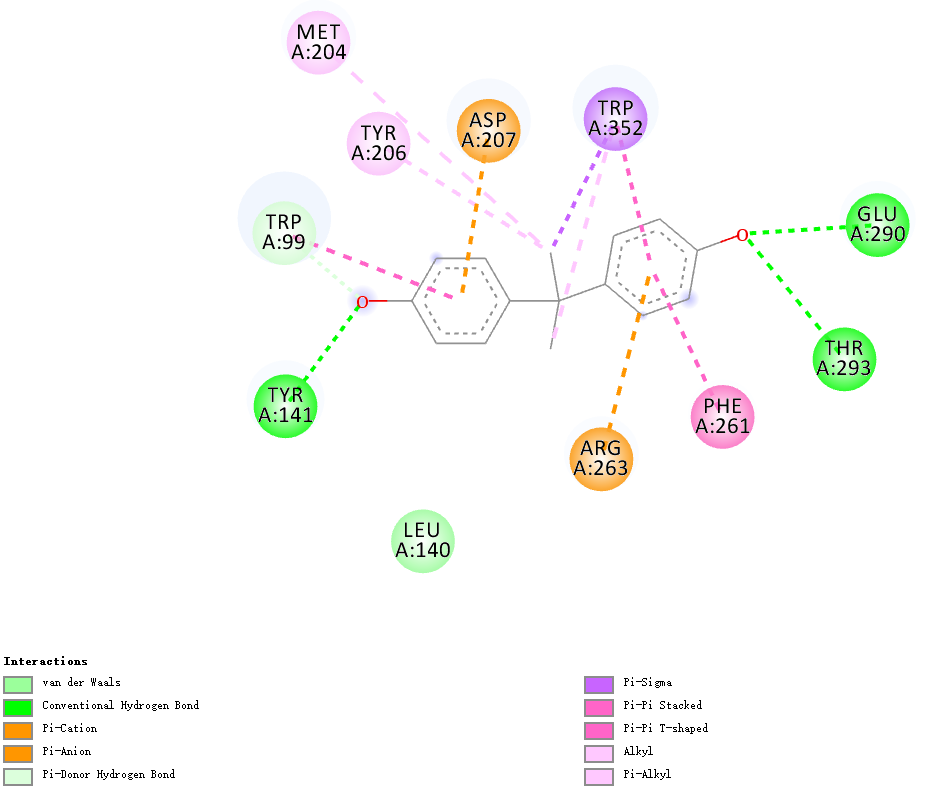

Supplement: Supplementary file 1 [file ijms-25-02504-s001.zip › ijms-2766183-supplementary/molecular docking/OASL/a3e0536807a239b0db39a7e00da038f.png]

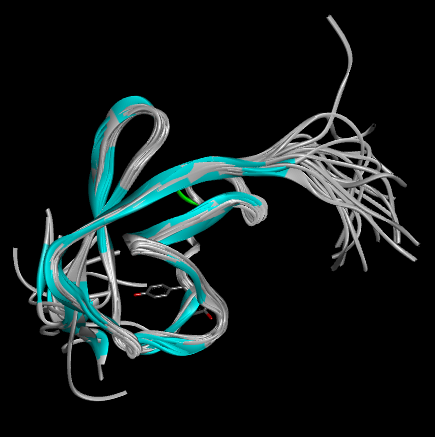

Supplement: Supplementary file 1 [file ijms-25-02504-s001.zip › ijms-2766183-supplementary/molecular docking/OASL/f54b05c64c35cab85b2e701cdad6430.png]

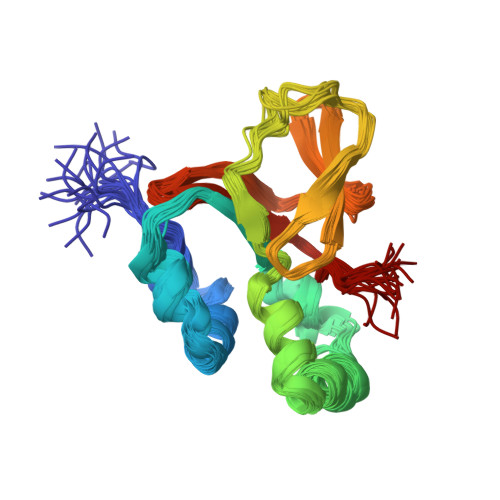

Supplement: Supplementary file 1 [file ijms-25-02504-s001.zip › ijms-2766183-supplementary/molecular docking/POSTN.1.jpeg]

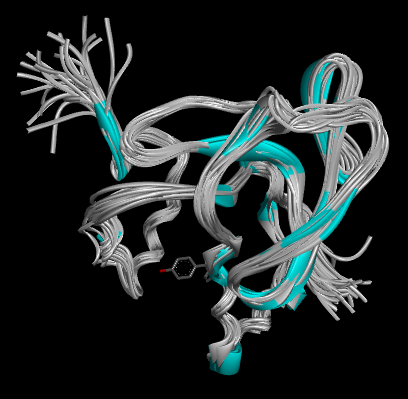

Supplement: Supplementary file 1 [file ijms-25-02504-s001.zip › ijms-2766183-supplementary/molecular docking/POSTN/282fa442c977f35f8560018b6070132.png]

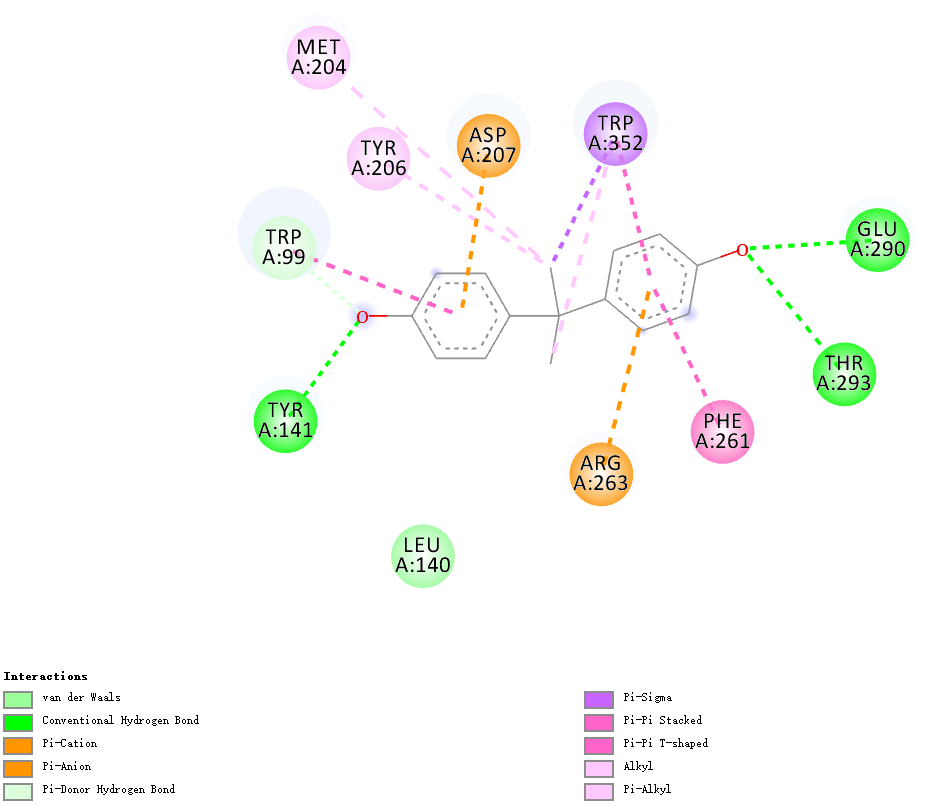

Supplement: Supplementary file 1 [file ijms-25-02504-s001.zip › ijms-2766183-supplementary/molecular docking/POSTN/c9281c11b00223466a0df2ac1c5f34a.png]

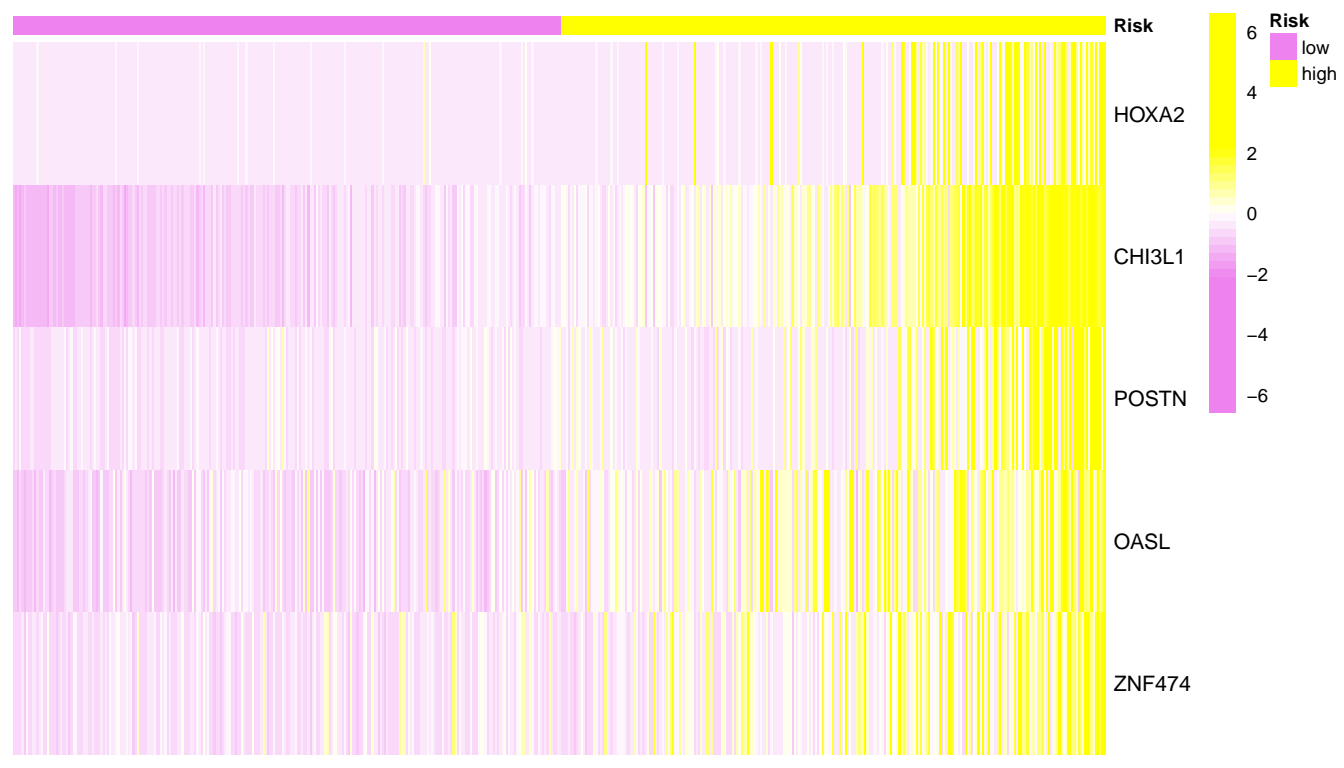

Supplement: Supplementary file 1 [file ijms-25-02504-s001.zip › ijms-2766183-supplementary/risk model/GEO.heatmap.pdf]

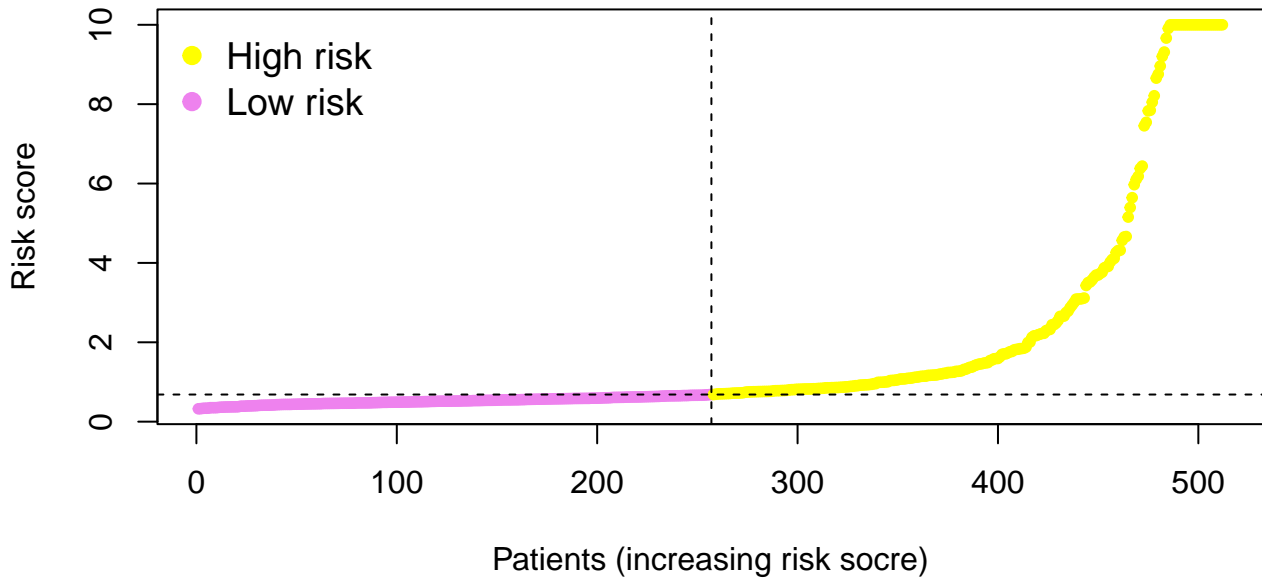

Supplement: Supplementary file 1 [file ijms-25-02504-s001.zip › ijms-2766183-supplementary/risk model/GEO.riskScore.pdf]

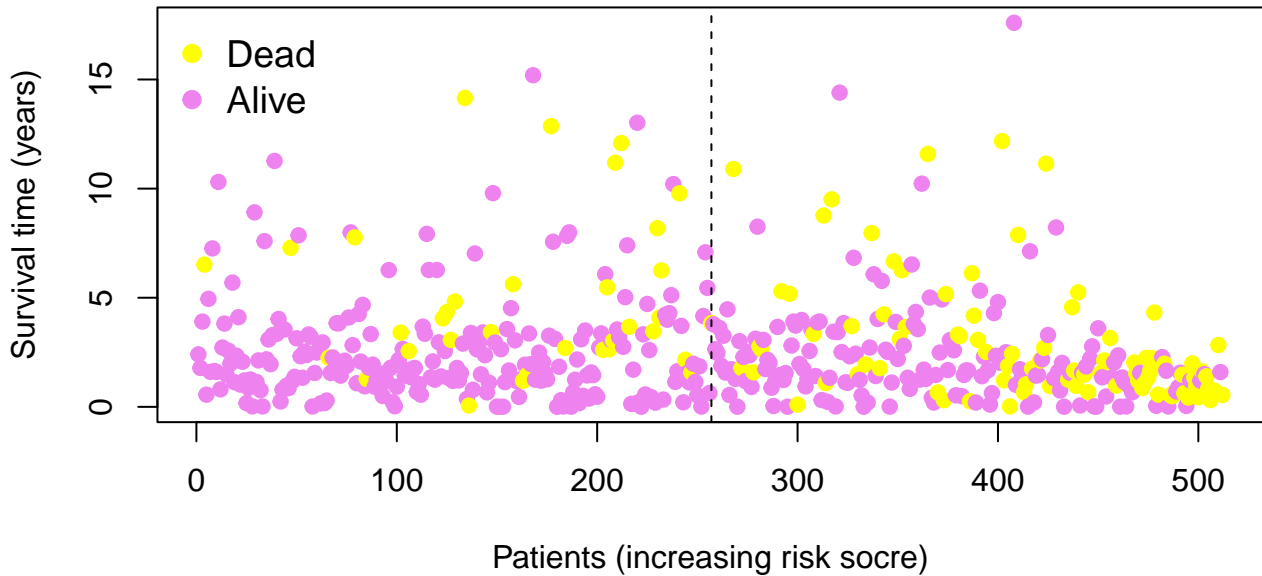

Supplement: Supplementary file 1 [file ijms-25-02504-s001.zip › ijms-2766183-supplementary/risk model/GEO.survStat.pdf]

Partial Likelihood Deviance

35 30 26 20 17 12 11 9 9 9 9 7 7 5 4 2 0

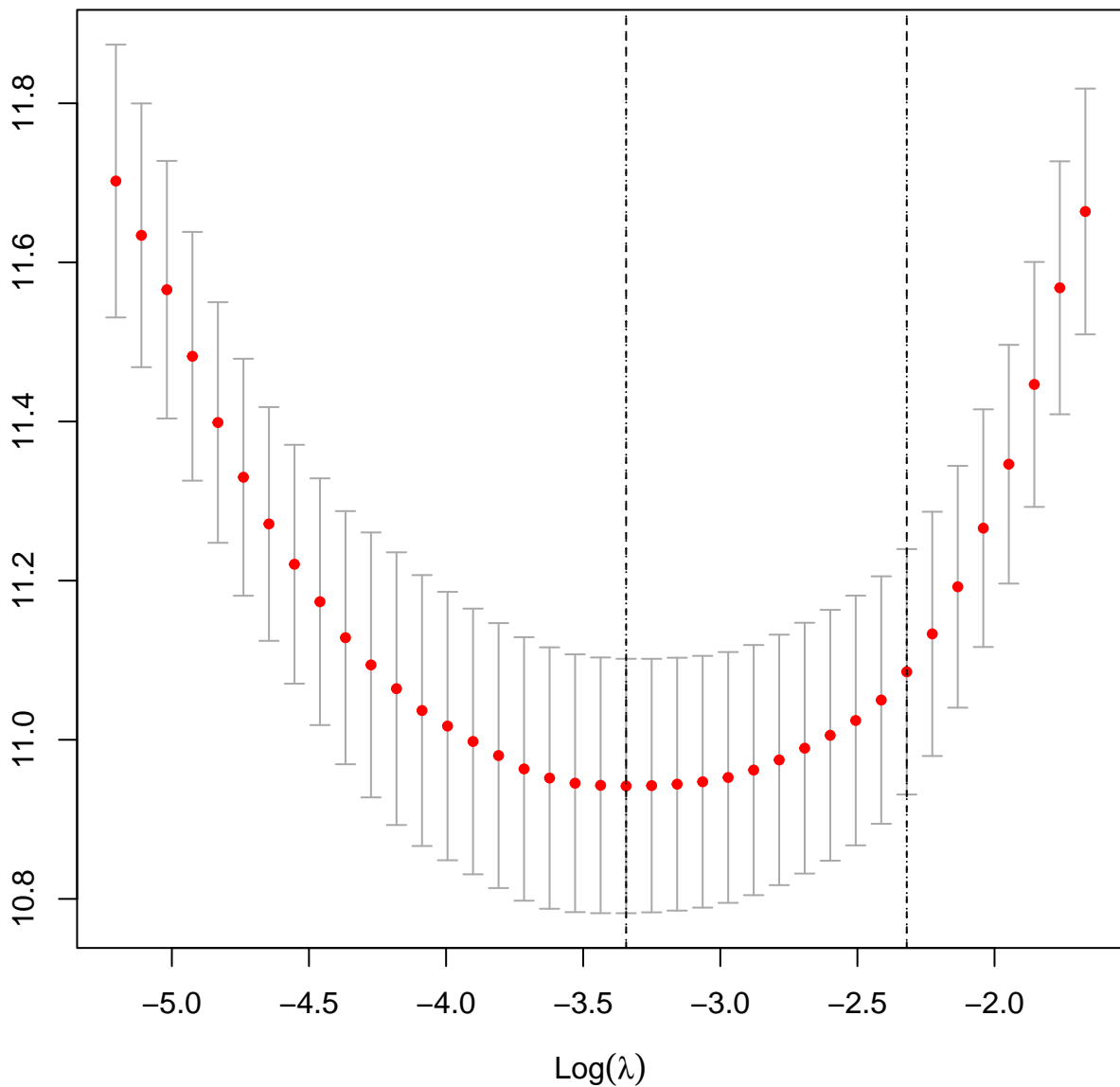

Supplement: Supplementary file 1 [file ijms-25-02504-s001.zip › ijms-2766183-supplementary/risk model/lasso.cvfit.pdf]

Coefficients

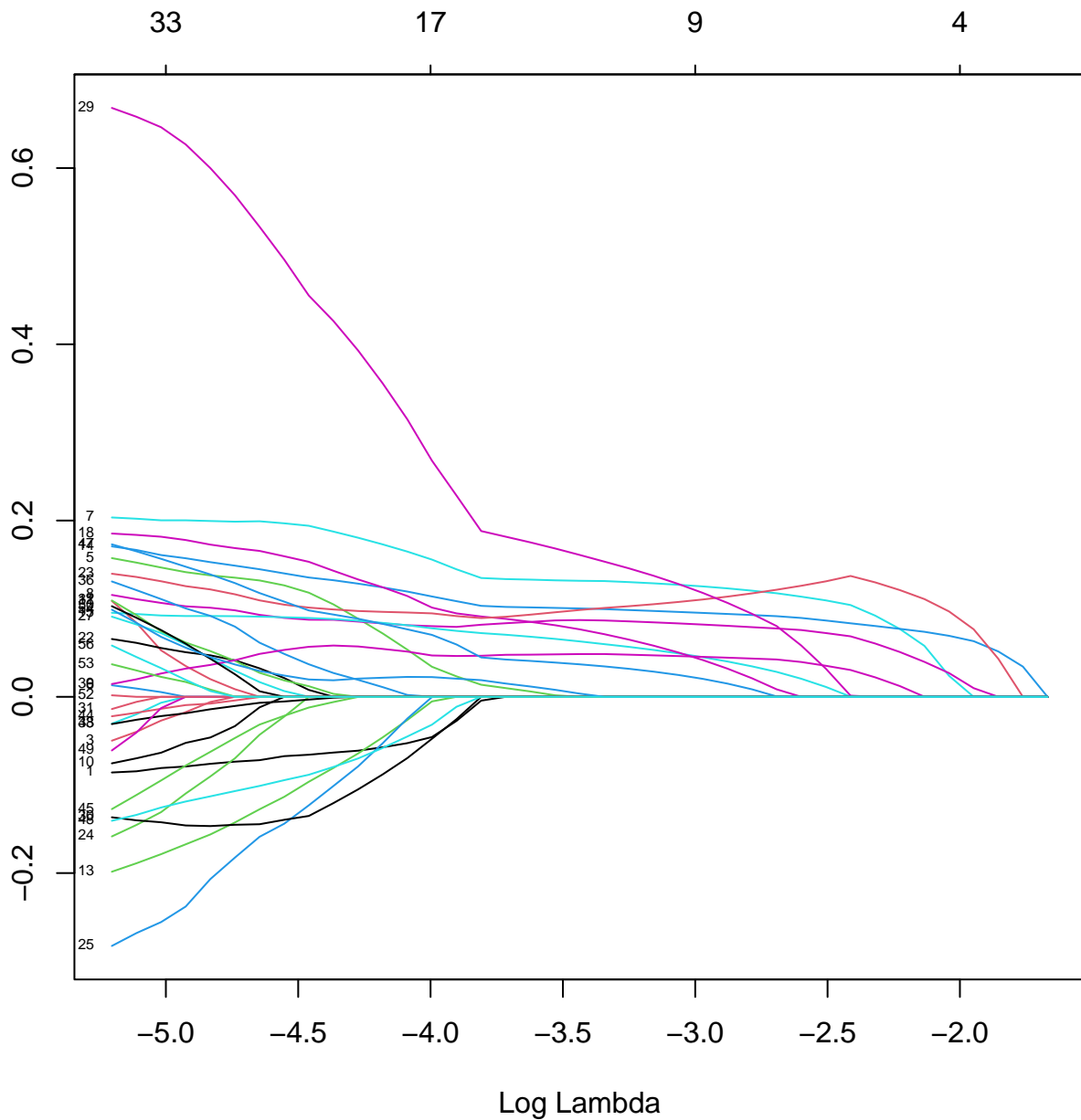

Supplement: Supplementary file 1 [file ijms-25-02504-s001.zip › ijms-2766183-supplementary/risk model/lasso.lambda.pdf]

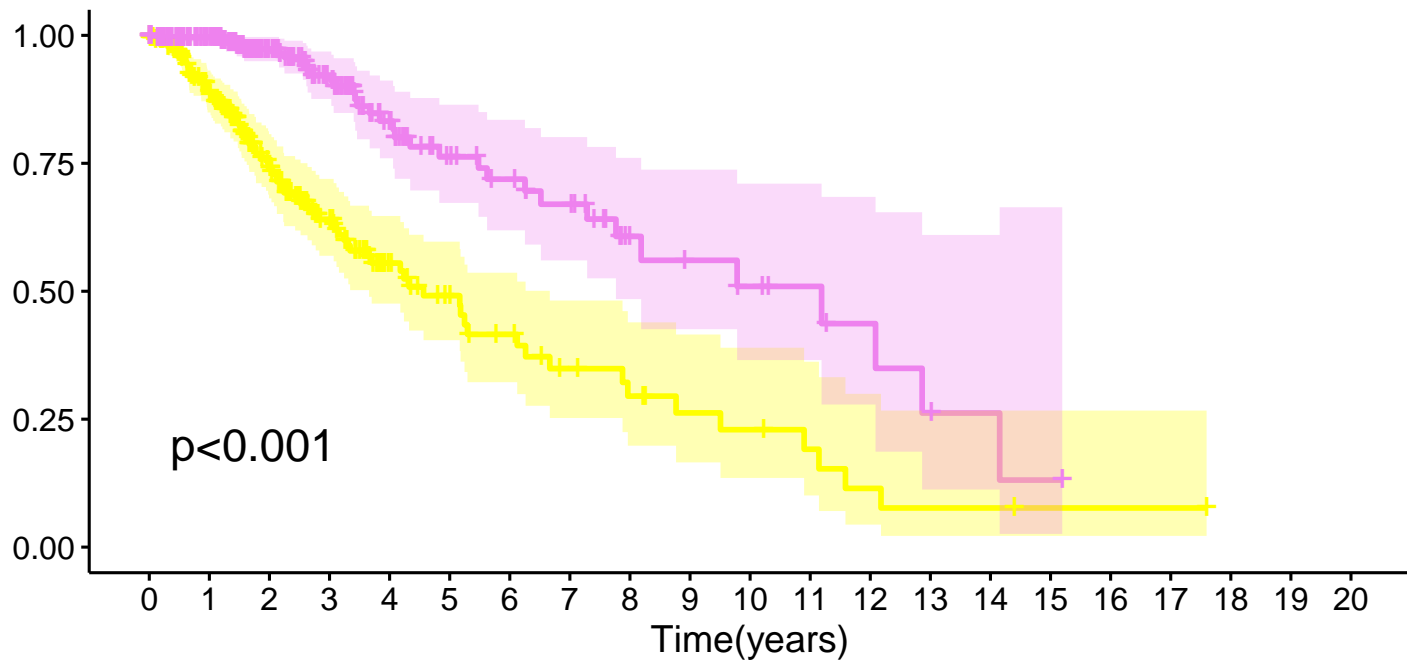

| Risk      | 0   | 1   | 2   | 3  | 4  | 5  | 6  | 7  | 8  | 9  | 10 | 11 | 12 | 13 | 14 | 15 | 16 | 17 | 18 | 19 | 20 |
|-----------|-----|-----|-----|----|----|----|----|----|----|----|----|----|----|----|----|----|----|----|----|----|----|
| High risk | 255 | 191 | 111 | 67 | 37 | 27 | 20 | 14 | 11 | 8  | 7  | 5  | 3  | 2  | 2  | 1  | 1  | 1  | 0  | 0  | 0  |
| Low risk  | 256 | 203 | 130 | 90 | 53 | 38 | 32 | 26 | 13 | 11 | 9  | 7  | 5  | 3  | 2  | 1  | 0  | 0  | 0  | 0  | 0  |

Supplement: Supplementary file 1 [file ijms-25-02504-s001.zip › ijms-2766183-supplementary/risk model/survival.GEO.pdf]

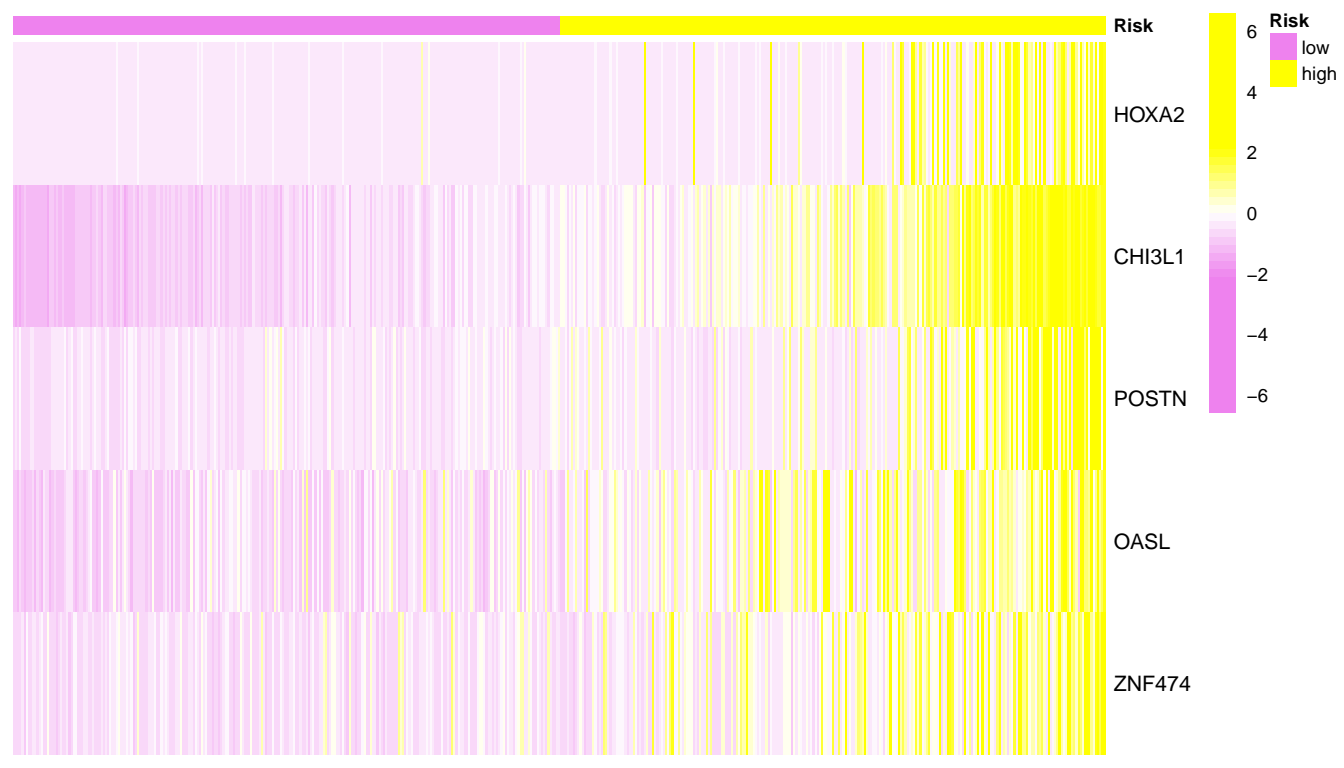

Supplement: Supplementary file 1 [file ijms-25-02504-s001.zip › ijms-2766183-supplementary/risk model/TCGA.heatmap.pdf]

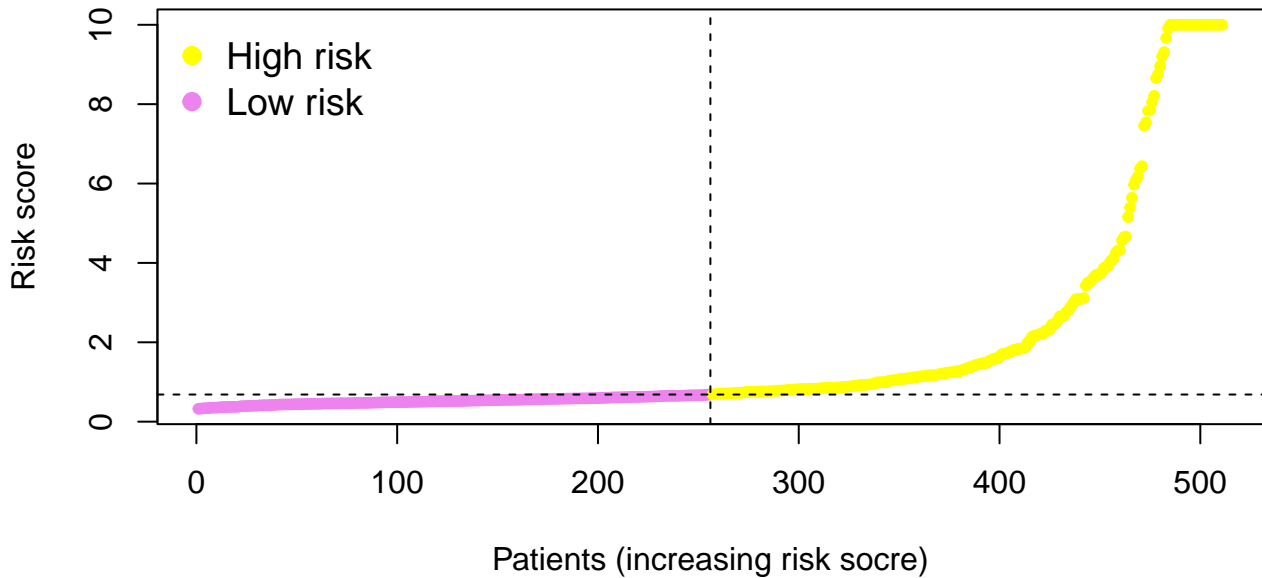

Supplement: Supplementary file 1 [file ijms-25-02504-s001.zip › ijms-2766183-supplementary/risk model/TCGA.riskScore.pdf]

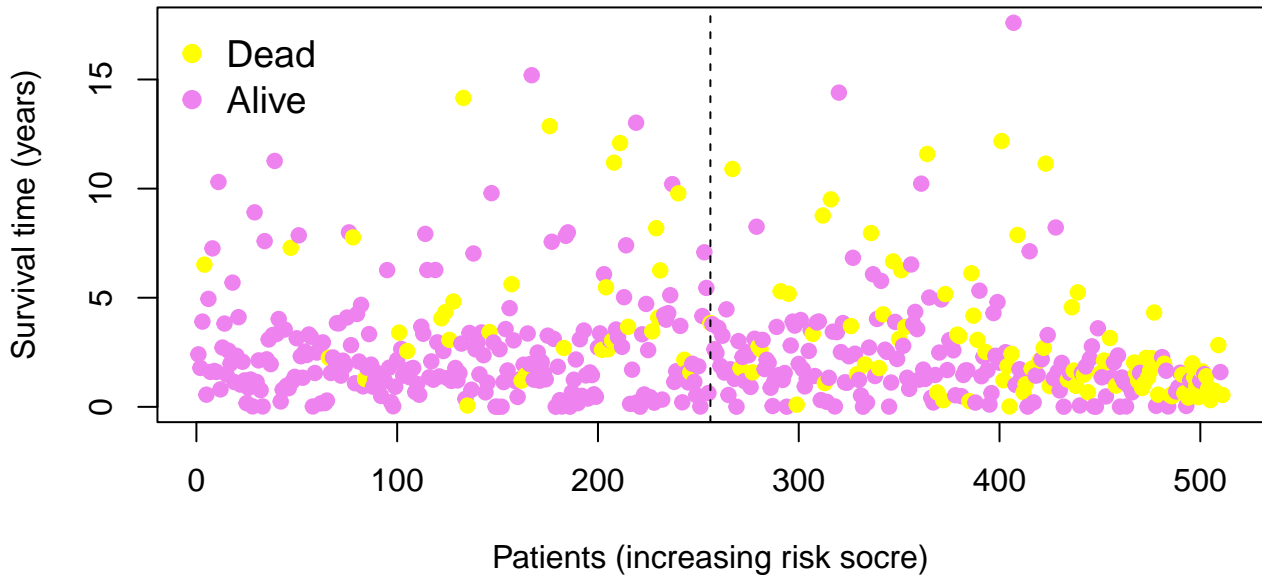

Supplement: Supplementary file 1 [file ijms-25-02504-s001.zip › ijms-2766183-supplementary/risk model/TCGA.survStat.pdf]

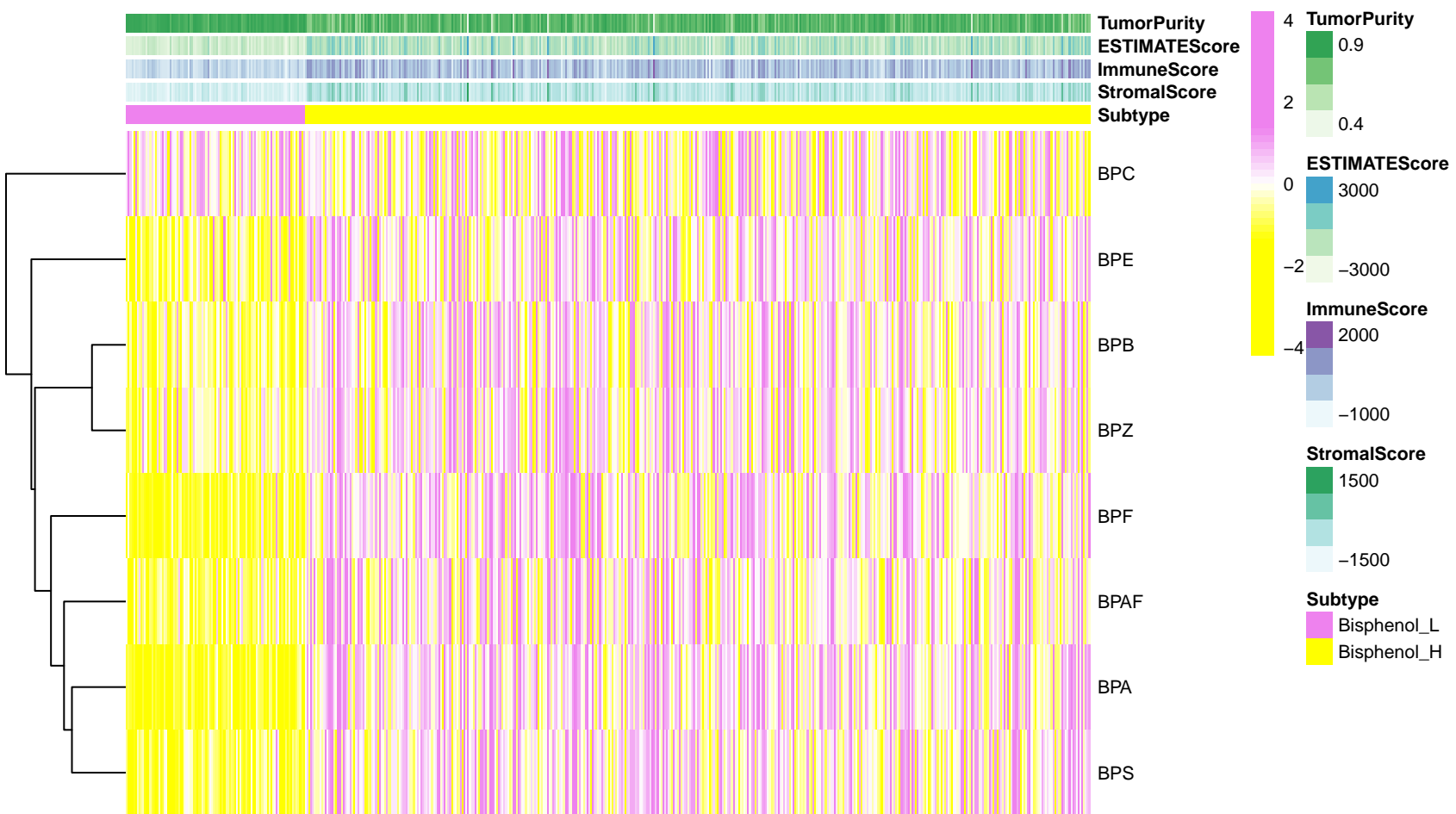

Supplement: Supplementary file 1 [file ijms-25-02504-s001.zip › ijms-2766183-supplementary/SSGSEA/estimateHM.pdf]

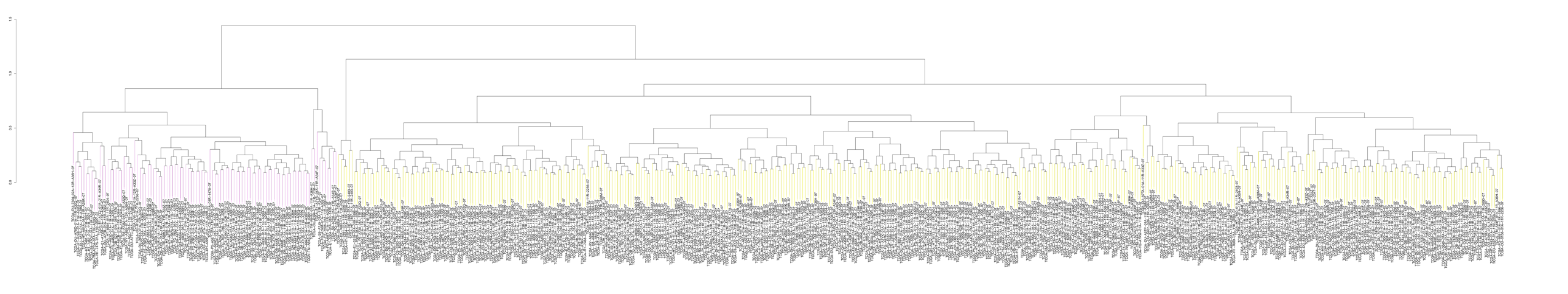

Supplement: Supplementary file 1 [file ijms-25-02504-s001.zip › ijms-2766183-supplementary/SSGSEA/hclust.pdf]

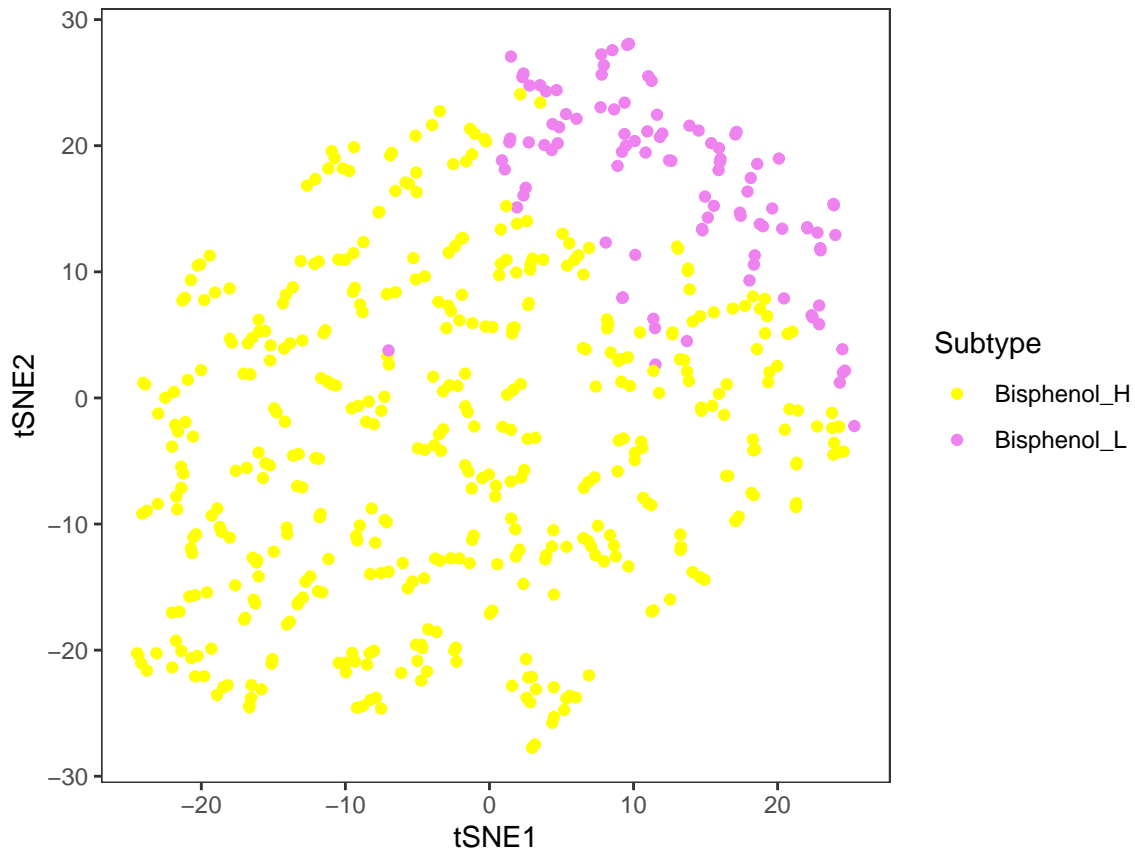

Supplement: Supplementary file 1 [file ijms-25-02504-s001.zip › ijms-2766183-supplementary/SSGSEA/tSNE.pdf]

Subtype 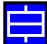 Bisphenol\_L 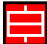 Bisphenol\_H

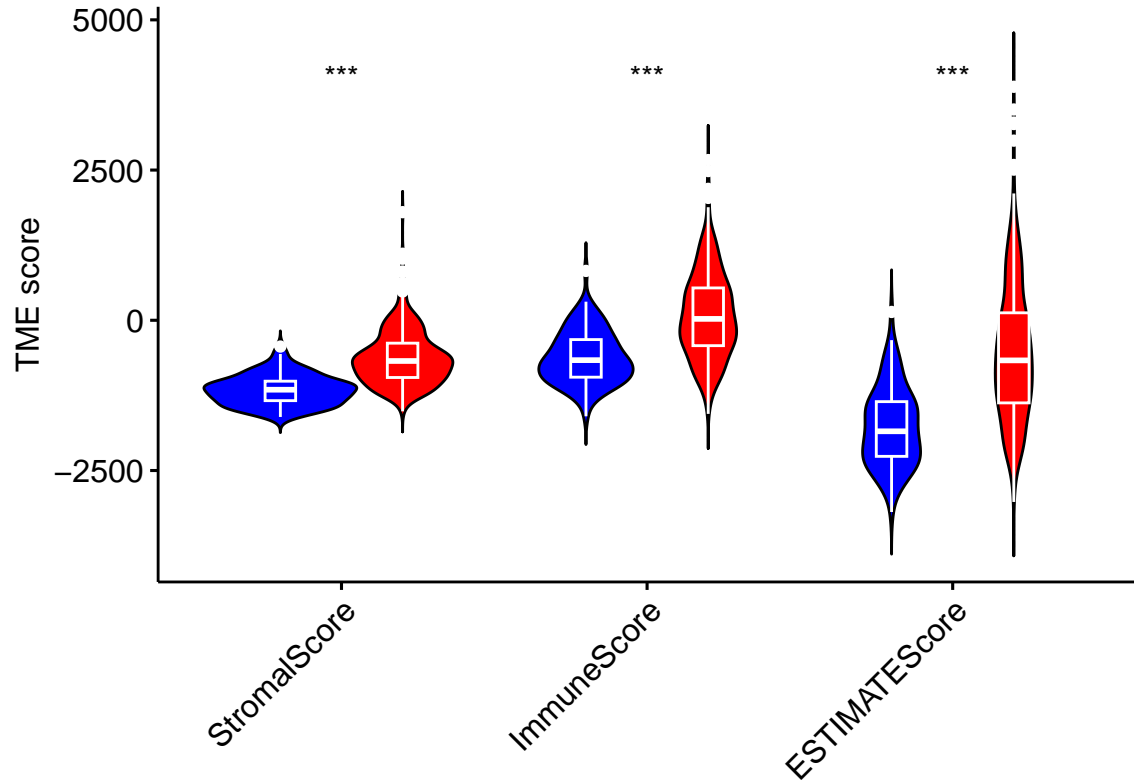

Supplement: Supplementary file 1 [file ijms-25-02504-s001.zip › ijms-2766183-supplementary/SSGSEA/vioplot.pdf]
