# Supplementary material for: Bisphenol A: Unveiling Its Role in Glioma Progression and Tumor Growth
Source: Int J Mol Sci. 2024 Feb 21;25(5):2504. doi: 10.3390/ijms25052504 (PMC10931199; doi:10.3390/ijms25052504)
Supplement: Supplementary file 1 [file Survival.CHI3L1.pdf › ijms-2766183-supplementary/after risk model/Survival.CHI3L1.pdf]

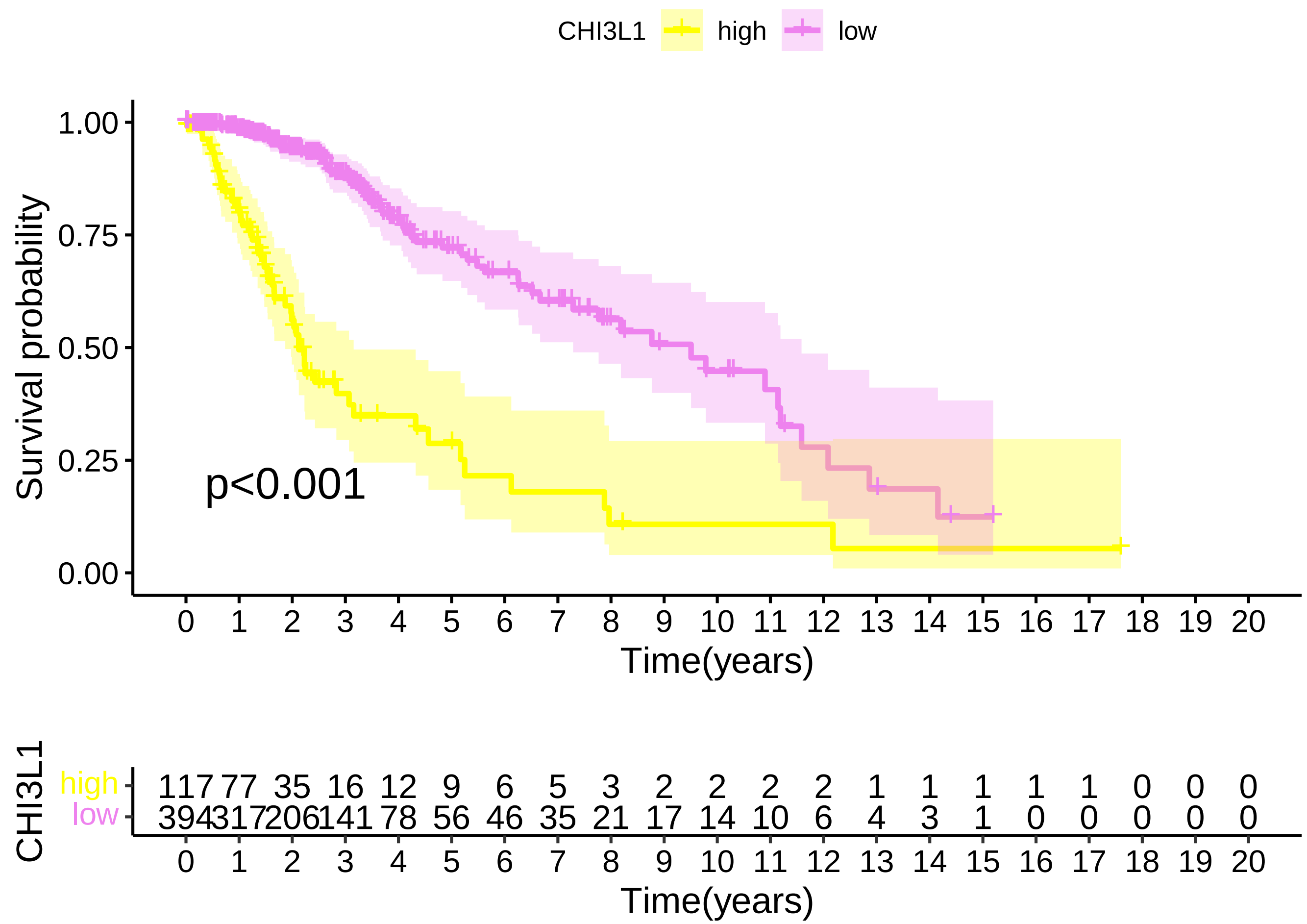
CHI3L1 high low
Survival probability
1.00
0.75
0.50
0.25
0.00
p<0.001
0 1 2 3 4 5 6 7 8 9 10 11 12 13 14 15 16 17 18 19 20
Time(years)
CHI3L1
high 117 77 35 16 12 9 6 5 3 2 2 2 2 1 1 1 1 1 0 0 0
low 394 317 206 141 78 56 46 35 21 17 14 10 6 4 3 1 0 0 0 0 0
0 1 2 3 4 5 6 7 8 9 10 11 12 13 14 15 16 17 18 19 20
Time(years)
